# Benchmarking of a microgel-reinforced hydrogel-based aqueous lubricant against commercial saliva substitutes

**DOI:** 10.1038/s41598-023-46108-w

**Published:** 2023-11-20

**Authors:** Olivia Pabois, Alejandro Avila-Sierra, Marco Ramaioli, Mingduo Mu, Yasmin Message, Kwan-Mo You, Evangelos Liamas, Ben Kew, Kalpana Durga, Lisa Doherty, Anwesha Sarkar

**Affiliations:** 1 Food Colloids and Bioprocessing Group, School of Food Science and Nutrition, University of Leeds, Leeds, LS2 9JT UK; 2 INRAE, AgroParisTech, UMR SayFood, Université Paris-Saclay, 91120 Palaiseau, France; 3 Unilever Research & Development Port Sunlight Laboratory, Bebington, CH63 3JW UK; 4 Vitrition UK Ltd, Liversedge, WF15 6RA UK; 5 ADM Protexin Ltd, Lopen Head, TA13 5JH UK

**Keywords:** Biophysics, Nanoscale materials, Soft materials, Materials science, Biomaterials, Bioinspired materials, Biomaterials - proteins

## Abstract

Xerostomia, the subjective sensation of ‘dry mouth’ affecting at least 1 in 10 adults, predominantly elders, increases life-threatening infections, adversely impacting nutritional status and quality of life. A patented, microgel-reinforced hydrogel-based aqueous lubricant, prepared using either dairy or plant-based proteins, has been demonstrated to offer substantially enhanced lubricity comparable to real human saliva in in vitro experiments. Herein, we present the benchmarking of in vitro lubrication performance of this aqueous lubricant, both in its dairy and vegan formulation against a range of widely available and employed commercial saliva substitutes, latter classified based on their shear rheology into “liquids”, “viscous liquids” and “gels”, and also had varying extensional properties. Strikingly, the fabricated dairy-based aqueous lubricant offers up to 41–99% more effective boundary lubrication against liquids and viscous liquids, irrespective of topography of the tested dry mouth-mimicking tribological surfaces. Such high lubricity of the fabricated lubricants might be attributed to their limited real-time desorption (7%) from a dry-mouth mimicking hydrophobic surface unlike the tested commercial products including gels (23–58% desorption). This comprehensive benchmarking study therefore paves the way for employing these microgel-based aqueous lubricant formulations as a novel topical platform for dry mouth therapy.

## Introduction

Xerostomia^1^ is clinically defined as the subjective complaint of dry mouth, which—due to a reduction and/or absence of salivary flow/lubricity^2^—results in oral friction and irritation. Affecting at least 1 in 10 adults, with rates as high as 30% in elders and 80% in institutionalised elders, oral dryness is one of the significant burdens on overall healthcare worldwide^3,4^. Over the past few years, the rising use of polymedication and cancer-related radiation therapies, and the increasing number of age-related chronic, neurogenerative, and autoimmune diseases (such as Sjögren's syndrome), combined with the dramatic growth of the global ageing population have been considered the main aetiologies of the global increase in xerostomia prevalence^5–7^. Dry mouth significantly increases the risk of dental caries, periodontal diseases, candidiasis, oral ulceration, and dysphagia, ultimately leading to reduced food intake and subsequent malnutrition^8–10^—all of which detrimentally and adversely impact the nutritional status, comfort, and overall quality of life of individuals. As a consequence, this symptomatic condition creates a heavy drain on healthcare resources (*i.e.*, longer hospital retention, and increased treatment cost and healthcare utilisation) and causes a tremendous economic burden^11^.

Although a very broad range and wide number of saliva substitutes (*i.e.*, symptomatic treatments mainly employed by xerostomia sufferers to rehydrate their oral cavity) exists in the marketplace, patients have largely reported their inefficacy and short-lived ability to alleviate symptoms, which force them to use their medical device repeatedly to be able to speak and eat, ultimately reducing the quality of life^12–16^. The key reason for such a sub-optimal performance is that these lubricants often contain thickening/ gelling agents (Table S1), which only provide viscous fluid film-based ‘hydrodynamic lubrication’^17,18^, and lack the biological surface adsorption-inducing ‘boundary lubrication’^19^ that is much needed in these high demanding lubrication failure situations. Although there has been significant progress in aqueous lubrication using biopolymers^20–23^ and polymeric hydrogel-based systems^24–26^, this dual benefit of boundary and hydrodynamic lubrication has not been completely achieved by any commercial or academic solutions, thereby leaving an unfilled knowledge gap in the market and scientific research. One proposed solution towards restoring oral hydration consists of designing new colloidal systems, as structured proteins- and polysaccharides-containing aqueous lubricants, which offer both boundary and hydrodynamic lubrication—a route that has remained poorly explored to date for dry mouth therapy.

**Figure 1:**
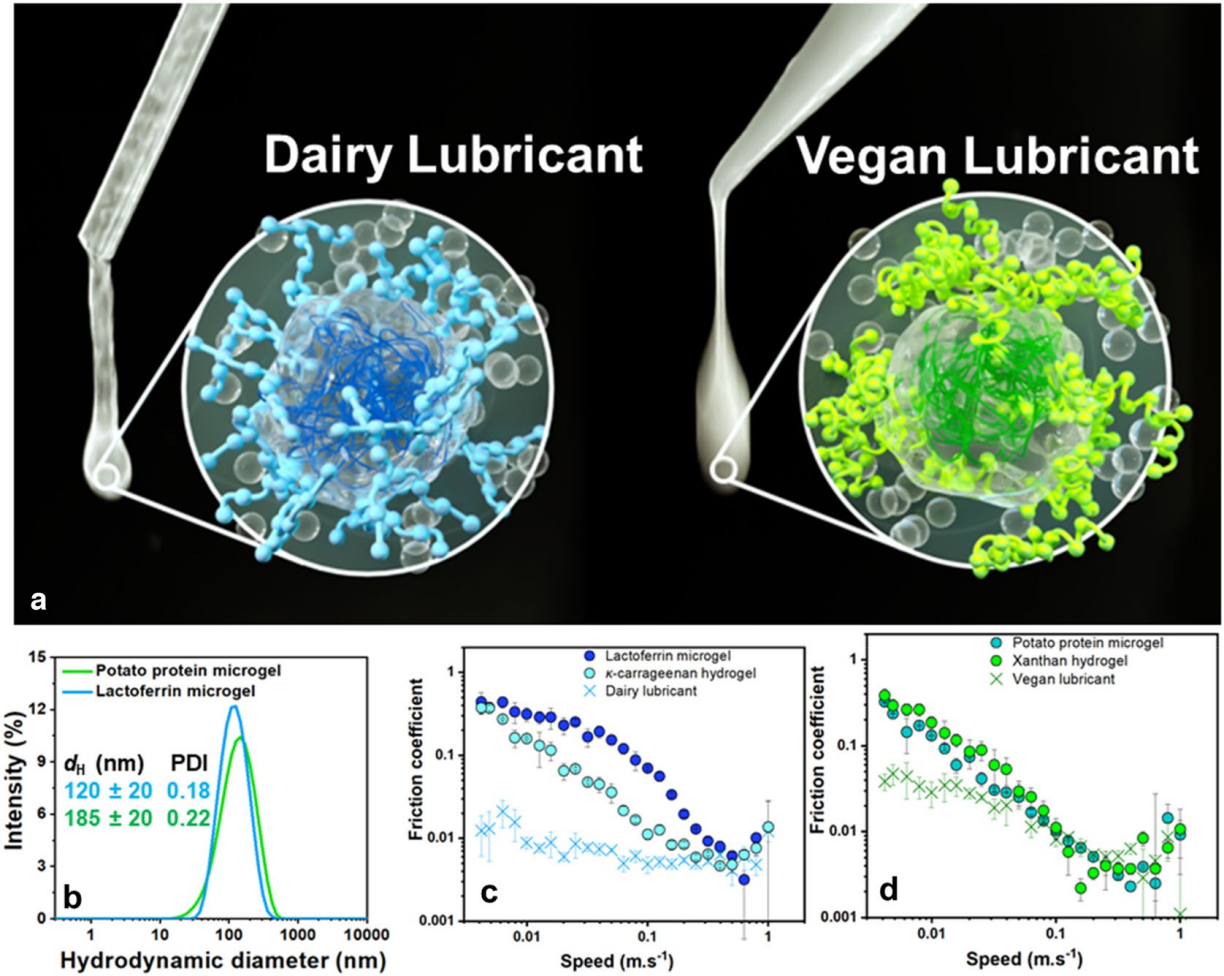
Illustration alongside properties of the fabricated aqueous lubricants. The visual gel-like image of the lab-made aqueous lubricants and their corresponding hypothetical molecular structure are shown in (**a**): based on electron microscopy data from previous literature^30^. The **left image** in **a** corresponds to the visual image of the dairy protein-based lubricant, with the zoomed schematic highlighting the dark blue lactoferrin within the grey mesh-like architecture of the hydrated lactoferrin-based microgel partially coated by the light blue *κ*-carrageenan-based hydrogel; the **right image** in** a** corresponds to the visual image of the vegan protein-based lubricant, with the zoomed schematic highlighting the dark green potato protein within the grey mesh-like architecture of the hydrated potato protein-based microgel partially coated by the light green xanthan gum-based hydrogel. The grey region represents the water phase. The particle size distribution with insets of hydrodynamic diameter (*d*_H_) and polydispersity index (PDI) of the microgels in the fabricated lubricants (**b**) and the lubrication properties of the dairy (**c**) and vegan (**d**) lubricants are also shown. In  (**c** and** d**), the boundary lubrication performance of the microgel-reinforced hydrogels exceeds that of their individual components (*i.e.*, the lactoferrin microgel and *κ*-carrageenan hydrogel in the case of the dairy lubricant at pH 7.0, and the potato protein microgel and xanthan gum hydrogel in the case of the vegan lubricant at pH 5.0). Each measurement was reproduced at least three times; the average measurement is shown and error bars represents standard deviations.

Human saliva, which is constituted of electrolytes and proteinaceous compounds (including large molecular weight (mucin) and small molecular weight (lactoferrin and amylase) proteins) dispersed in 99% water, plays a key role in oral functioning (speech, chewing, swallowing), providing oral tissues-protecting lubrication and promoting oral processing-facilitating food bolus formation, disintegration, and swallowing^27,28^. Its ability to coat and lubricate the oral cavity, ultimately preventing frictional damage, has recently been attributed to the synergistic interaction of electrostatically self-assembling salivary proteins: the negatively charged mucin forming a water-encapsulating mesh, thus enabling ‘hydrodynamic lubrication’, and the positively charged lactoferrin tethering the water reservoir to the surface, thus allowing ‘boundary lubrication’^29^. Building upon these fundamental insights into the nature-engineered salivary pellicle fabrication and its structurally induced lubrication mechanism, a colloidal formulation, composed of a proteinaceous microgel-reinforced biopolymeric hydrogel in a patchy architecture (see schematic illustration in Fig. 1), which offers substantially enhanced and sustained adsorption comparable to real human saliva, has been designed^30^ and patent has been filed^31^. However, the realisation of the full potential of this particular microgel-reinforced hydrogel based aqueous lubricant is stunted by the lack of a thorough benchmarking study against a range of commercial saliva substitutes such as sprays and gels.

Herein, we report a detailed investigation of the in vitro oral lubrication performance of this novel, colloidal aqueous microgel-reinforced hydrogel-based lubricant formulation made with dairy or vegan proteins (Fig. 1) benchmarked against a range of commercially available, sprays, viscous liquids and gel-type saliva-replacing products, using a complementary suite of rheological (shear and extensional), tribological and adsorption/ desorption measurements, where surfaces used were representative of dry mouth conditions. This work provides the first comprehensive evidence on the unrivalled capability of the microgel-based aqueous lubricant outperform as a saliva substitute based on high boundary lubrication and limited desorption properties. More specifically, the unprecedented results obtained from this thorough in vitro oral processing study show that the colloidal aqueous lubricant formulation fabricated using either a dairy or plant-based protein, and having higher viscosity than human saliva, drastically reduces boundary oral friction under in vitro dry mouth conditions to a much higher (41–99%) as compared to salivary substitutes that are thickened liquids and sprays. In particular, such high lubrication was attributed to the limited desorption (7%) than the entire range of commercial saliva-substituting products tested including (23–58% desorption) as well as high shear viscosity allowing fluid film lubrication—a key dual benefit making it unique over the competing brands. Such outperforming oral lubrication properties and outstanding retention capacity on in vitro biomimicking surfaces^32^ have been attributed to an optimal synergy between the two electrostatically binding components: the highly viscous, polysaccharide hydrogel generating ‘hydrodynamic lubrication’, and the efficiently adsorbing, proteinaceous microgel promoting ‘boundary lubrication’^30,31^. Therefore, this work offers the robust evidence for the potential of this new platform of colloidal microgel-based aqueous lubricants to work as a more efficient saliva substitute platform, which can be a game changer in the dry mouth therapy field and a life changing topical therapy for providing long lasting relief with ultra-lubricity to dry mouth sufferers, improving their quality of life.

## Results

### Brief description of the fabricated lubricants

Before benchmarking the fabricated lubricants against competitive salivary substitute samples, we briefly describe here the lab-fabricated aqueous lubricants prepared using dairy and vegetable proteins (henceforth referred to as dairy and vegan lubricants, respectively) (Fig. 1). As illustrated in Fig. 1a, the dairy lubricant contains a thermally cross-linked lactoferrin protein-based microgel that is partially coated by a* κ*-carrageenan hydrogel^30^, while the vegan lubricant is made up of a potato protein-based microgel and a xanthan gum hydrogel forming a patchy architecture (see the details of fabrication in the experimental section). Both lab-made aqueous lubricants exhibit a viscoelastic visual appearance and the microgels were sub-micron sized with low polydispersities (Fig. 1b) in line with previous report^30^. Of more importance, the study of their lubrication behaviour (Fig. 1c–d) shows a synergistic interaction between the proteinaceous microgel component and the polysaccharide-based hydrogel compound, as observed in our previous study^30^. In particular, the boundary friction coefficient was found to be of two orders of magnitude lower for the lactoferrin microgel-reinforced hydrogel (*i.e.*, dairy lubricant) (Fig. 1c) as compared to its individual components (*i.e.*, the lactoferrin microgel and *κ*-carrageenan hydrogel). For the vegan lubricant, the synergism was not as prominent as for the dairy lubricant. Nevertheless, the vegan lubricant demonstrated a boundary lubrication performance of an order of magnitude lower than its individual components (*i.e.*, potato protein microgel and xanthan gum hydrogel) (Fig. 1d).

### Classification based on viscous behaviour

**Figure 2:**
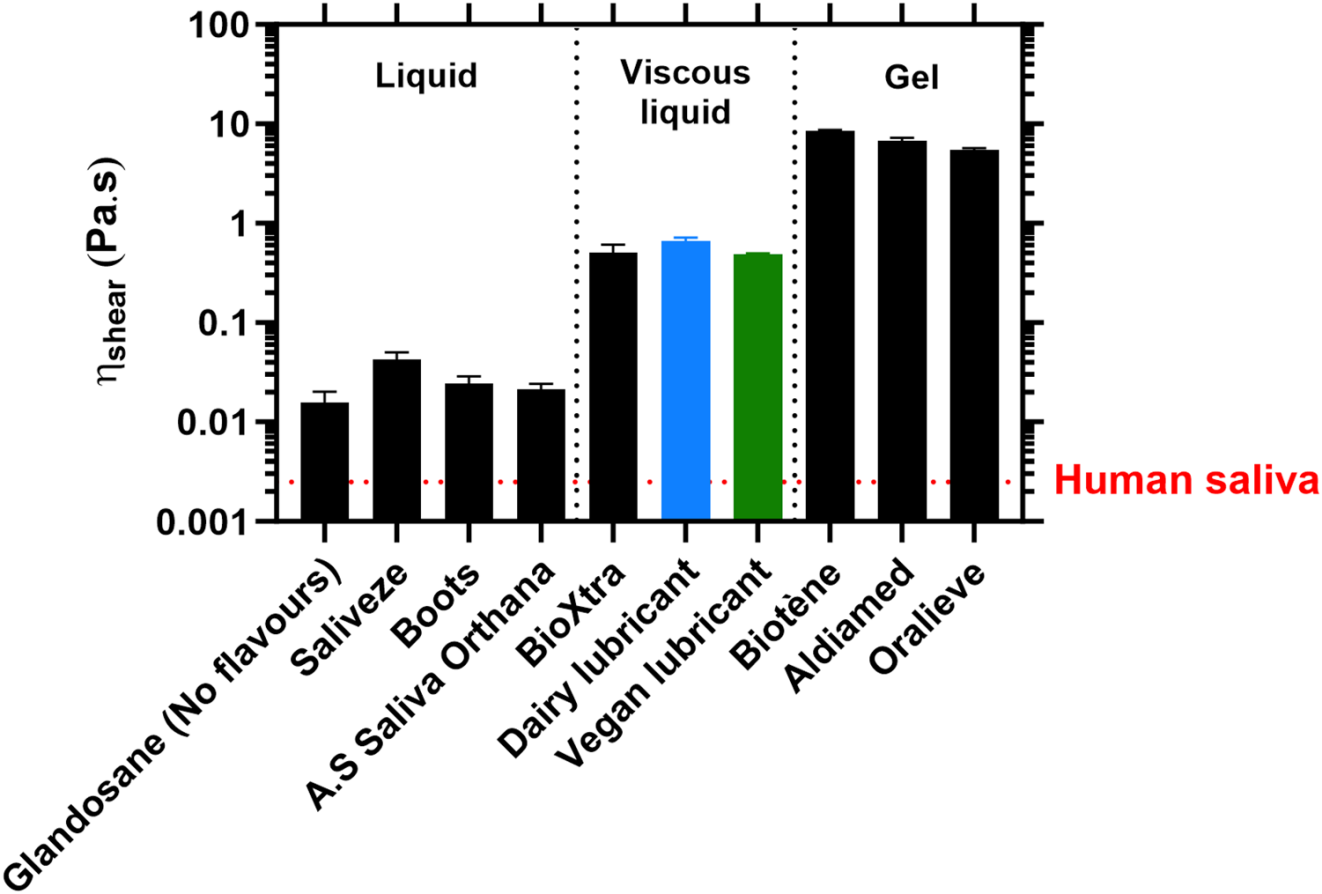
Shear viscosity of the fabricated aqueous lubricants benchmarked against commercial salivary replacers at an orally relevant shear rate. Comparison of the shear viscosity (*η*_*shear*_) obtained at an orally relevant shear rate (50 s^−1^)^33^, for the fabricated aqueous lubricant (both dairy and vegan alternatives) and a range of commercially available saliva substitutes (*liquids*: Glandosane (No flavours) from Fresenius-Kabi, Saliveze from Wyvern Medical, Boots, and A.S Saliva Orthana from CCMed; *viscous liquids*: BioXtra from RIS; and *gels*: Biotène from GSK, Aldiamed from Certmedica International, and Oralieve), at an orally relevant temperature (37 °C). These data were extracted from the flow curves measured using stress-controlled rotational rheometry measurements (Figure S1). The apparent shear viscosity of real human saliva (ca. *η*_shear_ = 2.5.10^–3^ Pa s) is also shown and used as control for comparison purposes^34^. Each measurement was reproduced at least three times; the average measurement is shown with error bars representing standard deviations. Samples were classified according to their shear viscosity (or viscous behaviour): the samples for which *η*_shear_ < 0.10 Pa s were termed ‘*liquids*’, *η*_shear_ = 0.10–1.0 Pa s ‘*viscous liquids*’, and *η*_shear_ > 1.0 Pa s ‘*gels*’.

The resistance to shear deformation of the two lab-fabricated, patented aqueous dairy and vegan lubricant formulations *vs*. existing salivary substitutes was measured to assess their viscous properties and classify them within rheologically-comparable groups (Figs. 2 and S1).

Shear viscosity measurements over a range of shear rates demonstrate a shear-thinning behaviour for the fabricated as well as all the commercial products studied, with nonetheless three different types of evolution: (1) Biotène, Aldiamed, and Oralieve exhibit a sharp (three orders of magnitude) decay in viscosity as the shear rate increases, and display similar shear viscosity values (*η*_shear_ = 8.4 ± 0.2 Pa s for Biotène, 6.7 ± 0.5 Pa s for Aldiamed, and 5.4 ± 0.2 Pa s for Oralieve, at 50 s^−1^) despite having distinct compositions (Table S1); (2) instead, Glandosane (No Flavours, Lemon flavour, and Peppermint flavour), A.S Saliva Orthana, Boots, and Saliveze show a one-to-two order of magnitude decrease in viscosity over the shear rate window, which reaches a near-plateau from ca. 1 s^−1^, and display strikingly lower shear viscosity values (*η*_shear_ = 0.012 ± 0.001 Pa s for A.S Saliva Orthana, *η*_shear_ = 0.016 ± 0.005 Pa s for Glandosane (No Flavours), η_shear_ = 0.025 ± 0.004 Pa s for Boots, *η*_shear_ = 0.041 ± 0.006 Pa s for Saliveze, at 50 s^−1^; (liquid *vs*. gels,* p* < *0.05*); (3) for BioXtra, the viscosity remains relatively constant at low shear rates, stabilising at ca. 1.6 Pa s, and starts decreasing slightly from 10 s^−1^, reaching *η*_shear_ = 0.548 ± 0.173 Pa s, at 50 s^−1^ (viscous liquid *vs*. liquids, *p* < *0.05*).

Despite differences in type of protein and polysaccharide types, both variants of the fabricated aqueous lubricant based on microgel-reinforced hydrogel exhibit very similar viscosity values (at 50 s^−1^, *η*_shear_ = 0.485 ± 0.015 Pa s and *η*_shear_ = 0.661 ± 0.058 Pa s, for the vegan and dairy lubricants, respectively (*p* > *0.05*)). Their flow curves exhibit the same profile as those of Biotène, Aldiamed, and Oralieve, thus showing a clear pseudo-plastic behaviour, with nonetheless shear viscosity values comparable to those displayed by BioXtra, at orally relevant shear rates.

Based on these rheological measurements, the commercial salivary substitute products including the fabricated aqueous lubricants studied were classified in three different formulation categories: (1) *liquids* for the products whose *η*_*shear*_ values are below 0.10 Pa s, at the orally relevant shear rate (50 s^−1^) (*i.e.*, Glandosane (No Flavours, Lemon flavour, and Peppermint flavour), A.S Saliva Orthana, Boots, and Saliveze); (2) *viscous liquids* for *η*_*shear*_ values ranging between 0.10 and 1.0 Pa s, at 50 s^−1^ (*i.e.*, BioXtra and both the alternatives of the fabricated aqueous lubricant); and (3) *gels* for *η*_*shear*_ values being above 10 Pa s, at 50 s^−1^ (*i.e.*, Biotène, Aldiamed, and Oralieve).

A preliminary assessment of the sensory perception of these samples highlighted that *liquids* and *viscous liquids* exhibited, respectively, a water consistency and coating feeling, whereas *gels* were sensed as sticking to the tongue and oral surfaces (data not shown). Human saliva has a remarkably low viscosity (ca. *η*_shear_ = 2.5.10^–3^ Pa s^34^), close to that of water (ca. *η*_shear_ = 1.0.10^–3^ Pa s). Of note, all samples, including the *liquids*, had *η*_*shear*_ values higher than that of saliva (*p* < *0.05*). The classification based on this rheological characterisation (Fig. 2) is used henceforth to compare the tribological, adsorption, and extensional properties of the samples.

### Lubrication performance based on friction-reducing effects

**Figure 3:**
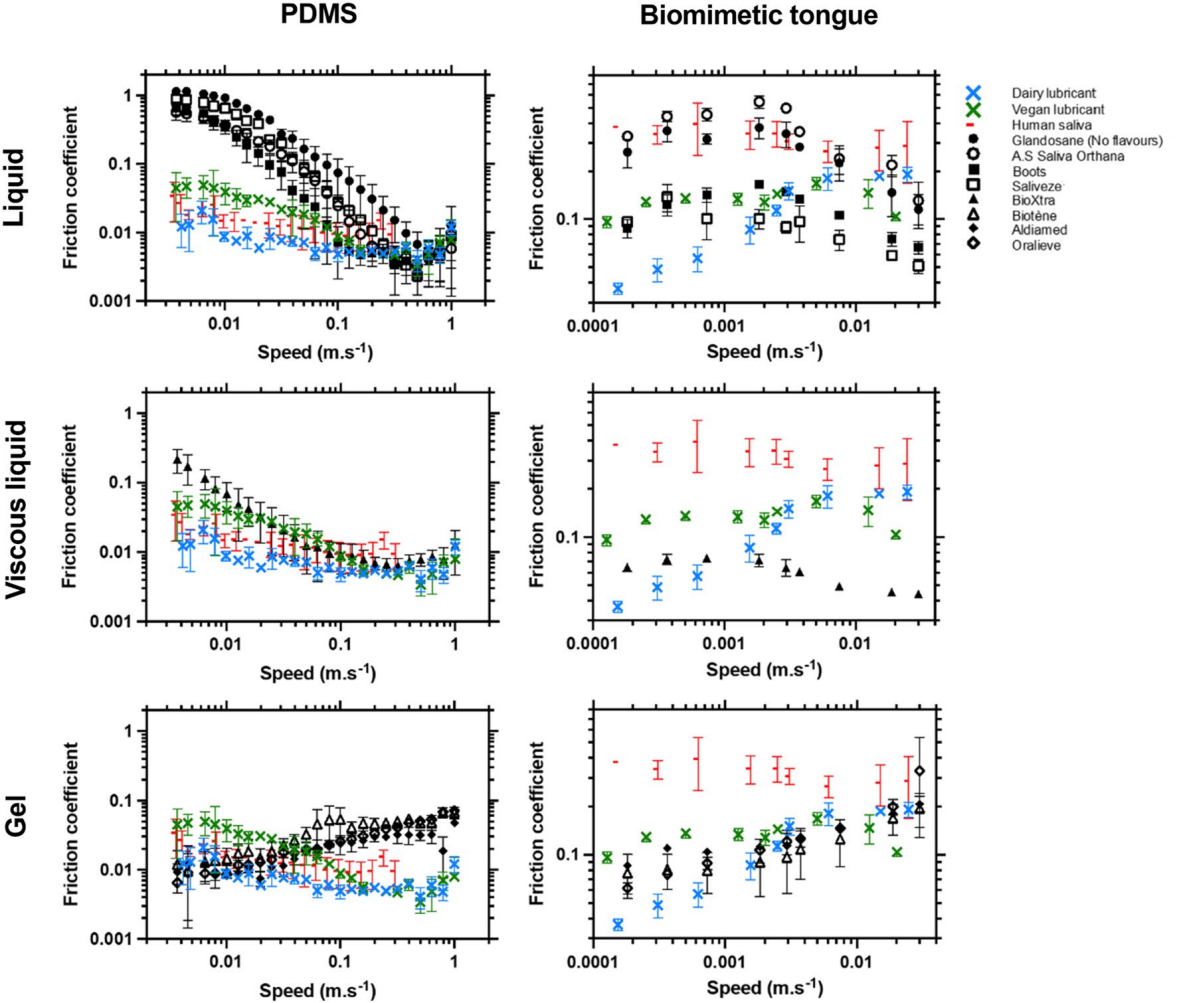
Lubrication performance of the fabricated aqueous lubricant benchmarked against commercial salivary replacers under orally relevant conditions. Speed-dependent evolution of the friction coefficient, obtained from tribology measurements performed with **i|** smooth (PDMS), and **ii|** 3D-textured, biomimetic tongue-like surfaces replicating a dry mouth, on the fabricated aqueous lubricant (both dairy and vegan alternatives) and a range of commercially available saliva substitutes: ***liquids:*** Glandosane (No flavours) from Fresenius-Kabi, A.S Saliva Orthana from CCMed, Boots, and Saliveze from Wyvern Medical (see Figure S2 for additional liquids); ***viscous liquids:*** BioXtra from RIS; and ***gels:*** Biotène from GSK, Aldiamed from Certmedica International, and Oralieve, at an orally relevant temperature (37 °C). The lubrication properties of real human saliva are also shown and used as controls for comparison purposes (MEEC 16–046 ethics approved by the Faculty Ethics Committee, University of Leeds). Each measurement was reproduced at least three times; the average measurement is shown with error bars representing standard deviations. The fabricated dairy lubricant shows an outstanding lubrication performance in the boundary regimes, exhibiting much lower friction coefficients than the commercial *liquid* and *viscous liquid* samples irrespective of the type of surfaces (smooth vs textured). The boundary lubricity of the fabricated lubricants is similar to that of real human saliva in the presence of PDMS surfaces, and significantly lower than saliva in the presence of a biomimetic tongue surfaces. Unlike dairy, the vegan lubricant showed comparable friction as compared to some commercial viscous liquids (see details on speed dependent comparison in Figure S3) and higher friction than viscous liquids in biometic tongue-like surfaces.

The ability of the fabricated aqueous lubricants (either the dairy or vegan alternative) to reduce oral friction and ultimately protect the oral cavity was studied by tribology, using dry mouth-mimicking surfaces, and compared with that of saliva substitutes currently available on the marketplace (Fig. 3, Figures S2 and S3). In this study, tribology measurements were conducted using conventional, smooth, highly hydrophobic elastomeric (PDMS) surfaces, which are commonly used in oral lubrication studies. In addition, a more biologically relevant 3D-textured elastomeric surface replicating a real human dry tongue surface in terms of topography, upper bound of contact pressures (ca. 130 kPa), and hydrophobic character^32^, where the 3D biomimetic tongue-like surface focused on boundary speed limits^35^. Although, both the surfaces had similar surface hydrophobicity (115.0° ± 1.0° for Smooth PDMS and 112.0° ± 10.0° for textured elastomer, *p* < *0.05*) ^32^ they differed in contact pressures, speeds and consequently varied in film thickness of the lubricants tested. Thus, using two different surfaces^19,30^ with varying topography and contact pressures offer complementary understanding to the benchmarking of the fabricated lubricants against the commercial salivary substitutes as well as represent a wide range of dry tongue surfaces that range from completely de-papillated to a tongue with significant number of papillae^36,37^.

Tribology measurements usually show three regimes, which—in the case of oral lubrication^38^—represent three different stages of food oral processing, and different amounts of lubricant in the mouth: (i) at high speeds, a large amount of lubricant is entrained in between the two surfaces (replicating here a tongue and palate) and forms a layer that bears the load and pushes the surfaces apart, ultimately reducing friction—in the hydrodynamic regime, the ability to decrease friction depends on the lubricant rheology; (ii) at low speeds, the two surfaces are in contact with each other, therefore excluding the sample from the contact area, and resulting in high friction coefficients—this boundary regime is strongly influenced by the lubricant ability to adsorb onto the substrates; (iii) in between these two regions is the mixed regime, for which both wetting and viscous lubrication play a key role in friction reduction.

Corroborating the rheological results, three lubrication behaviours can also be distinguished when comparing the commercial saliva substitutes (Fig. 3): (1) *liquids* (*i.e.*, Glandosane (No Flavours, Lemon flavour, and Peppermint flavour), Saliveze, Boots, and A.S Saliva Orthana) show similarly shaped frictional curves to buffer^39^ (data not shown), exhibiting particularly high friction coefficients in the boundary region, exceeding 0.36 ± 0.08 at 0.01 m s^−1^ with PDMS surfaces, and 0.10 ± 0.03 at 0.0007 m s^−1^ with the biomimetic tongue surface; (2) *viscous liquids* (*i.e.*, BioXtra) generate much lower friction coefficients than *liquids* at low speeds, in particular reaching 0.07 ± 0.03 at 0.01 m s^−1^ with PDMS surfaces, and 0.07 ± 0.01 at 0.0007 m s^−1^ with the biomimetic tongue surface; and (3) compared to *liquids* and *viscous liquids*, *gels* (*i.e.*, Biotène, Aldiamed, and Oralieve) provide lower friction coefficients in the boundary regime, where friction coefficient values as low as 0.01 ± 0.01 and 0.08 ± 0.02 are obtained, respectively, at 0.01 m s^−1^ with PDMS surfaces and at 0.0007 m s^−1^ with the biomimetic tongue surface, therefore demonstrating a better efficacy at lubricating oral surfaces than *liquid* and *viscous liquid* products (liquid *vs*. gel, *p* < *0.05*). This is consistent with observations made previously with an ex vivo, reciprocating sliding tongue-enamel system, with which several saliva substitutes (including A.S Saliva Orthana spray, Glandosane spray) were shown to poorly enhance oral lubrication and ultimately relieve dry mouth symptoms^19^.

Focusing now on the fabricated aqueous lubricants, tribology measurements in the presence of PDMS surfaces (Figure S3) demonstrate that both the dairy and vegan alternatives drastically reduce the friction between the two surfaces in contact, both in the boundary (reaching 0.01 ± 0.01 and 0.04 ± 0.01, at 0.01 m s^−1^, for the dairy and vegan variants, respectively) and hydrodynamic (reaching 0.01 ± 0.01, at 0.25 m s^−1^, for both variants) regions, performing strikingly better than the marketed products tested (the *liquids* and *viscous liquids* exhibiting friction coefficients exceeding 0.07 ± 0.03 at 0.01 m s^−1^. The dairy lubricant lowered friction more than those of naturally lubricating human saliva (which displays friction coefficients of 0.02 ± 0.01, at both 0.01 and 0.25 m s^−1^) (*p* < *0.05*) irrespective of speeds. The dairy lubricant outperformed the gels (*p* < *0.05*) particularly in the hydrodynamic regime. The vegan lubricant, however, showed a more sporadic behaviour as compared to the dairy lubricant when comparing to the saliva and the gels. The vegan lubricant shows friction equivalent to that of saliva (*p* > *0.05*) in low-to-medium speeds (0.01–0.05 m s^−1^), but lower friction than saliva in the higher speeds (0.25 m s^−1^) (*p* < *0.05*). In addition, the vegan lubricant shows sporadic behaviour with higher friction coefficients than gels in boundary lubrication regime (*p* < *0.05*) in presence of PDMS, equivalent friction to gels in medium speeds (0.05 m s^−1^) (*p* < *0.05*), but lower friction than gels in the hydrodynamic regime (0.04 ± 0.01 at 0.25 m s^−1^, *p* > *0.05*).

The tribology experiments involving the biologically relevant 3D-textured tongue-like surface show a slightly different behaviour (Fig. 3). The relatively high friction induced by real human saliva while in contact with the biomimetic tongue surface (*i.e.*, 0.39 ± 0.14 at 0.0007 m s^−1^) could be explained by the dilution process in the sample preparation, resulting in a relatively lower surface-adsorbing protein content. One can question, that such high frictional behaviour of saliva was not apparent in PDMS-PDMS contact surfaces. This may be attributed to the difference in surface topography and contact pressure where a sample dilution might result in limited drag force to overcome the surface asperities of hundreds of micron levels, unlike a smooth PDMS surface where roughness is < 50 nm. The fabricated dairy lubricant displays unprecedented lubrication properties that remarkably exceed those of both existing products and real human saliva, exhibiting particularly low friction coefficient values at 0.0007 m s^−1^ in biomimetic tongue-like surface (friction coefficients = 0.06 ± 0.01 for the dairy variant, *vs*. friction coefficients > 0.07 ± 0.01 for commercial saliva substitutes and friction coefficients = 0.39 ± 0.14 for human saliva, *p* < *0.05*). One signature feature was the dairy lubricant behaved similar to those of gels in presence of biomimetic tongue like surface showing early onset of elastohydrodynamic lubrication at very low speeds < 0.001 m s^−1^, in other words, viscous-dominated separation between surfaces despite having an order of magnitude lower shear viscosity than gels (*p* < *0.05*) (Fig. 2).

For vegan lubricant on the other hand, the friction is lower than saliva and most commercial sprays (Figure S2) (*p* < *0.05*) but equivalent to Boots, and Saliveze (*p* > *0.05*) in presence of biomimetic tongue-like surface (Fig. 3). Although the vegan lubricant had better entrainment in the surface asperities and consequently demonstrated lower friction than saliva (*p* < *0.05*), it did not outperform the lubricity of the viscous liquids and gels (Fig. 3) in the lower speeds. Of more importance, unlike the fabricated dairy lubricant and gels, the vegan lubricant had a speed-independent behaviour in lower speed regimes (< 0.001 m s^−1^) in the biomimetic tongue-like surface similar to that of saliva profile and did not show rapid onset of elastohydrodynamic regime. Taken altogether, these results highlight the high boundary lubrication potential of the fabricated technology particularly the dairy-based lubricant particularly against the liquids and viscous liquids and comparable behaviour to those of gels despite having lower viscosity, irrespective of the surfaces used.

### In vitro hydration behaviour based on adsorption/desorption properties

**Figure 4:**
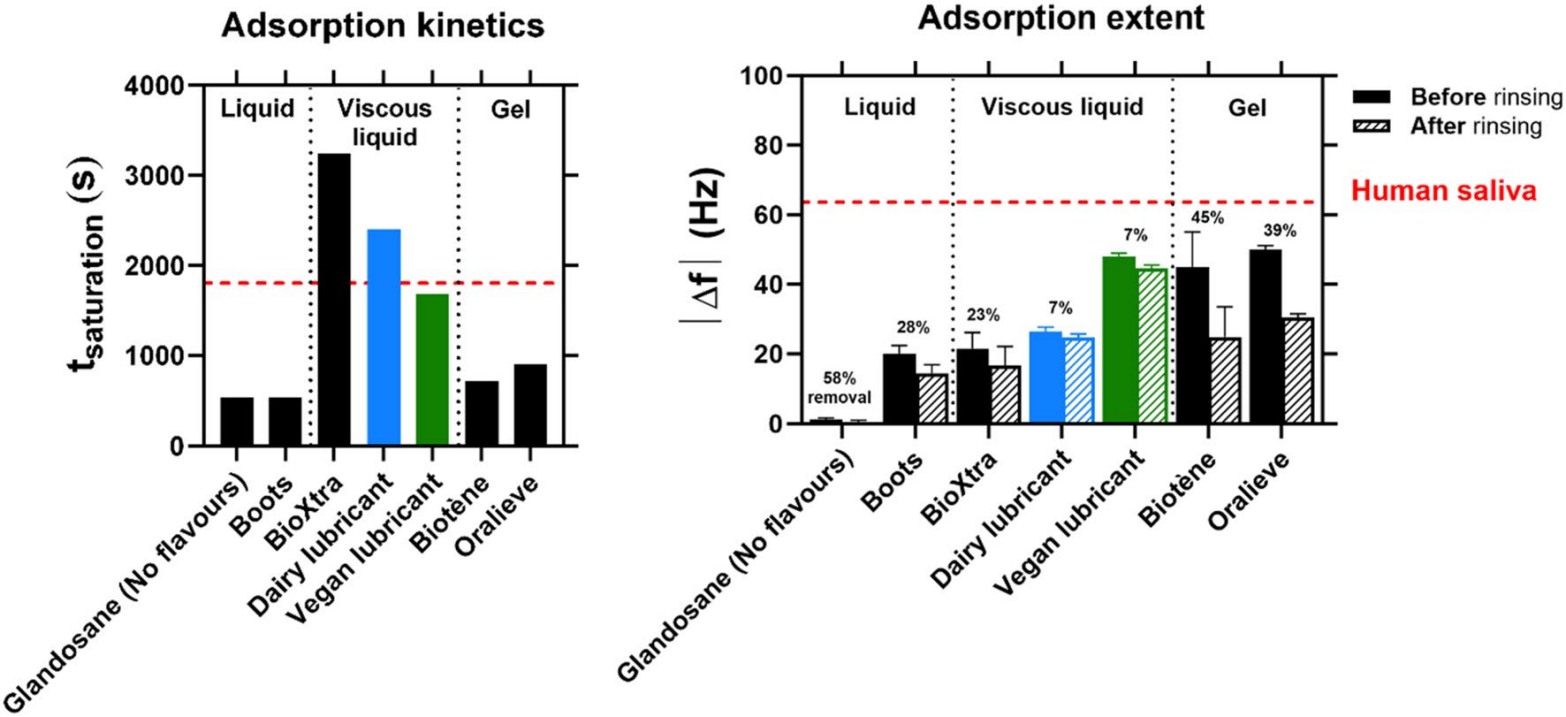
Hydration capacity of the fabricated aqueous lubricant benchmarked against commercial salivary replacers, in the presence of a dry mouth-mimicking surface. Comparison of the time required to reach adsorption saturation (*t*_*saturation*_) before rinsing, which is characteristic of the adsorption kinetic, and the resonance frequency (Δ*f*) reached before and after rinsing, which is an indication of the adsorption extent, obtained for the fabricated aqueous lubricant (both dairy and vegan alternatives) and a reduced range of commercially available saliva substitutes (*liquids*: Glandosane (No flavours) from Fresenius-Kabi, Saliveze from Wyvern Medical, Boots, and A.S Saliva Orthana from CCMed; *viscous liquids*: BioXtra from RIS; and *gels*: Biotène from GSK, Aldiamed from Certmedica International, and Oralieve), in the presence of a dry mouth-replicating, PDMS-coated surface. These data were extracted from the frequency curves measured using quartz crystal microbalance with dissipation monitoring measurements (Figure S4). The proportion of material removed, or the desorption extent, is also indicated and calculated considering the decrease in resonance frequency (Δ*f*) following rinsing; this parameter is used here as an in vitro indicator of the stickiness index of the samples, higher values corresponding to lower stickiness indexes. The adsorption properties of real human saliva are also shown and used as controls for comparison purposes (MEEC 16-046 ethics approved by the Faculty Ethics Committee, University of Leeds). These data were extracted from the QCM-D measurements (Figure S4). Each measurement was reproduced at least three times; the average measurement is shown with error bars representing standard deviations. The fabricated aqueous lubricants take time to adsorb at the interface similar to that of saliva, particularly the vegan alternative. More importantly, unlike commercial salivary substitutes, fabricated lubricants remain strongly attached following rinsing, therefore exhibiting interfacial properties similar to those of real human saliva, which efficiently adsorbs at the interface and barely undergoes desorption.

Besides lubricity, substantial and sustained adhesion of the lubricant film or so-called “coating” or “hydration” of the residual mucosal layer, or inner epithelium completely devoid of any remnant mucosal layer in the extreme case of dry mouth, is crucial to providing long-lasting mouth moisturising properties and to limiting the need and repeated use of a medical device. Such coating of the lubricant to the disrupted mucosal layer may overcome the transient nature of the current dry mouth therapies and offer increased longevity and relief period^12^ to the enabling dry mouth sufferers. The capacity of the fabricated aqueous lubricant *vs.* marketed salivary substitutes to adsorb onto a hydrophobic, dry mouth-emulating, PDMS-coated silica substrate, following ingestion (*i.e.*, following lubricant injection into the chamber) and swallowing (*i.e.*, following buffer injection into the chamber), was studied using a quartz crystal microbalance with dissipation (QCM-D). Based on tribology measurements, which have shown that products from the same shear rheology category decrease friction following a relatively similar trend, a reduced number of commercial samples was selected for this in vitro longevity assessment study. The evolution of the resonance frequency (Δ*f*), which correlates with the adsorbed mass^40^, was followed over time (Figure S4). The saturation time (*t*_*saturation*_), corresponding to the time required to reach maximum adsorption upon lubricant addition, and the resonance frequency (Δ*f*) obtained before and after rinsing, which is proportional to the quantity of material adsorbed following adsorption and desorption, respectively, are shown in Fig. 4. In this work. we conceptually define the proportion of lubricant removed from the interface upon rinsing as the “in vitro coating index”, such that increased desorption means reduced coating.

Independently of the lubricant type, a decrease in resonance frequency (Δ*f*) was observed following its addition into the chamber, indicating lubricant adsorption onto the PDMS-coated support, while the injection of buffer was found to result in an increase in resonance frequency, implying the desorption of material upon rinsing (Figure S4). Results show that both *liquids* (*i.e.*, Glandosane (No Flavours) and Boots) and *gels* (*i.e.*, Biotène and Oralieve) adsorb relatively quickly at the interface, with Δ*f* plateauing at its maximum value after a *t*_*saturation*_ ranging between 9 and 15 min, whereas *viscous liquids* (*i.e.*, BioXtra) and the fabricated aqueous lubricant (both alternatives) were found to require more time to reach adsorption saturation (ca. *t*_*saturation*_ = 54 min for BioXtra, and ca. *t*_*saturation*_ = 40 and 28 min for the dairy and vegan alternatives, respectively), a behaviour similar to that of real human saliva (ca. *t*_*saturation*_ = 30 min).

In term of adsorption extent, *gels* (*i.e.*, Biotène and Oralieve) were shown to adsorb readily on the PDMS-coated surface, achieving high | Δ*f* | values (| Δ*f* |= 45.1 ± 9.9 and 50.0 ± 1.2 Hz, respectively) relatively close to that displayed by human saliva (| Δ*f* |= 63.6 ± 2.5 Hz). Instead, *liquids* (*i.e.*, Glandosane (No Flavours) and Boots) and *viscous liquids* (*i.e.*, BioXtra) seemed to interact less efficiently with the PDMS solid interface, reaching much lower | Δ*f* | values following saturation (| Δ*f* |= 21.6 ± 4.6 Hz for BioXtra, | Δ*f* |= 20.6 ± 2.3 Hz for Boots, and only | Δ*f* |= 1.3 ± 0.5 Hz for Glandosane (No Flavours)) (Glandosane (No Flavours) *vs*. vegan lubricant and gel, *p* < *0.05*). When comparing both variants of the fabricated lubricant, the vegan version was found to attach to the surface to a higher extent than the dairy one (| Δ*f* |= 48.0 ± 1.0 Hz *vs*. 26.4 ± 1.4 Hz for the vegan alternative *vs*. dairy alternative).

Buffer rinsing caused the desorption of a significant amount of material from the interface in the case of Glandosane (No Flavours) *liquid*, Biotène *gel*, and Oralieve *gel*, which underwent a 39% to 54% removal, thus showing poor in vitro coating indices (Glandosane (No Flavours) *vs*. gels, *p* < *0.05*). Boots *liquid* and BioXtra *viscous liquids* were found to lose only 28% and 23% of their hydration layer, respectively, upon rinsing. The fabricated aqueous lubricant displayed the lowest proportion of material desorbed (7% of removal proportion for both the dairy and vegan alternatives) (Glandosane (No Flavours) *vs*. fabricated aqueous lubricants, *p* < *0.05*), thus exhibiting an in vitro coating index closely resembling that of human saliva for which desorption barely occurs (4% removal). An oral tribological study involving an ex vivo system shows comparable results of limited coating of some of these commercial saliva substitutes tested in the current study in a different tongue/enamel set-up and confirms the short relief period in vivo displayed by these saliva replacers offering seconds to minutes long short relief periods^12^. To summarize, the key benefit offered by the fabricated lubricant irrespective of the formulation is the limited desorption (7%) versus all other competitive products (23–58%) upon rinsing, which might offer longer retention and needs to be investigated in the future using in vitro and in vivo temporal studies.

### Extensional behaviour

**Figure 5:**
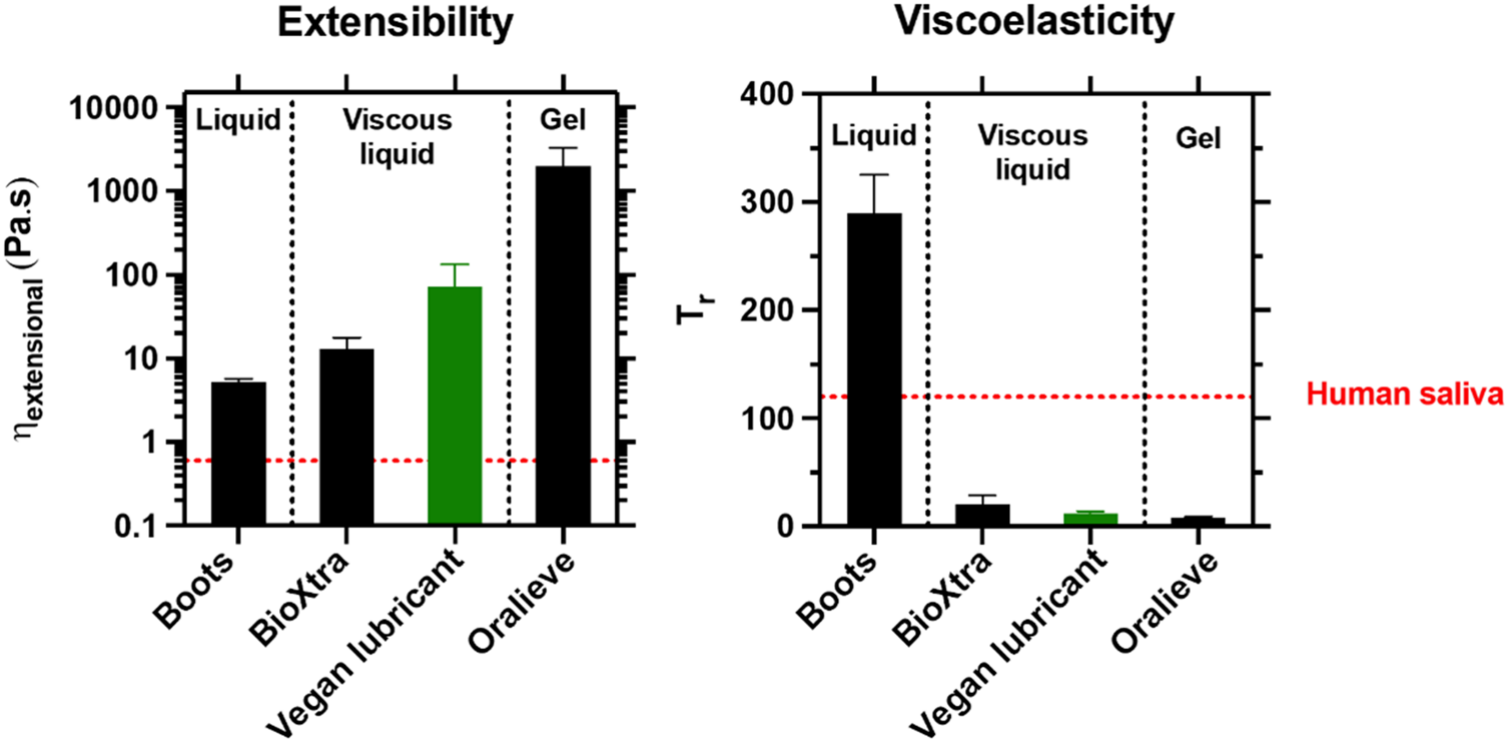
Extensibility and viscoelasticity of the fabricated vegan aqueous lubricant benchmarked against commercial salivary replacers. Comparison of the maximum apparent extensional viscosity (*η*_*extensional*_) (*i.e.*, extensibility) and Trouton ratio (*T*_*r*_) (*i.e.*, viscoelasticity) obtained for the fabricated aqueous lubricant (vegan alternative) and a reduced range of commercially available saliva substitutes (*liquid*: Boots; *viscous liquid*: BioXtra from RIS; and *gel*: Oralieve), at an orally relevant temperature (37 °C). These data were extracted from the extensional rheometry measurements (Figures S6 and S7). It is worth noting that, given the very wide range of rheological properties of the samples studied, computing the trouton ratio considering a constant value of shear viscosity would introduce important biases. The dependence of the shear viscosity on the strain rate has therefore been considered. As a result, the maximum apparent extensional viscosity and trouton ratio are not necessarily reached simultaneously, nor at the same strain. Some non-homogeneity was observed for the vegan lubricant, which reduces the repeatability and probably also the accuracy of the extensional measurements. The rheological properties of real human saliva (ca. *η*_extensional_ = 0.6 Pa s and T_r_ = 120) are also shown and used as controls for comparison purposes^48^. Each experiment was reproduced at least three times; the average measurement is shown with error bars representing standard deviations.

While the stretchiness of saliva has recently been proven to play a key role in preventing food bolus elongation/breakage and subsequent residue aspiration during the swallowing phase^41–47^, the importance of extensional properties in other oral functionalities, such as mastication (*i.e.*, food bolus formation and processing) and speech articulation, remains elusive. Despite these uncertainties, the resistance to stretching of the fabricated aqueous lubricant formulation *vs*. existing saliva substitutes was measured on a capillary breakage extensional rheometer to complete the overall picture on benchmarking of fabricated lubricants against salivary substitutes based on robust material characterization. Because products from the same format group were found to display similar lubrication and adsorption properties, the extensibility of only one product per shear rheological category from Fig. 2 was assessed. Changes in capillary thread shape (Figure S6) and diameter (Figure S7) upon extensional deformation were recorded over time; the maximum apparent extensional viscosity (*η*_*extensional*_) and Trouton ratio (*T*_*r*_, which is characteristic of the lubricant viscoelasticity) obtained through the fitting of these measurements with the models described by Eqs. (1) and (2) (see method section for the equations) are shown in Fig. 5.

For all the samples measured, the filament thinning mechanism observed can be separated into two stages: (i) an initial regime, where a long thread forms, (ii) followed by a fast decay rapidly evolving into an axially uniform thin filament, eventually breaking up (Figures S6 and S7). Nonetheless, similarly to rotational rheology measurements, extensional rheology experiments highlight clear discrepancies in stretchiness between commercial salivary replacers from different format categories: (1) *liquids* (*i.e.*, Boots) create thin and slender, filaments, which do not withstand extension over a long period of time (t_b_ = 0.03 ± 0.003 s); (2) on the contrary, *gels* (*i.e.*, Oralieve) were found to form very long-lived capillary threads much more resistant to extensional deformation, breaking up at a time of t_b_ = 24.25 ± 13.72 s; (3) compared to *gels* and *liquids*, *viscous liquids* (*i.e.*, BioXtra) show an intermediate behaviour, resisting filament thinning for t_b_ = 0.31 ± 0.01 s. Even though exhibiting a larger capillary filament diameter, the vegan version of the fabricated aqueous lubricant follows a thread formation mechanism similar to BioXtra *viscous liquid*, not breaking up before t_b_ = 1.63 ± 1.21 s. The thinning dynamics of Boots *liquid* was captured well by the elastic model (Eq. (1)), while that of *viscous liquids* and *gels* were better described by the power law model (Eq. (2)). The cylindrical shape of the Boots filaments is coherent with the dominant elastic properties and beads-on-a-string instabilities have been observed (Figure S6) and the shape of the other filaments (Figure S6) coherent with a power law shear viscosity model.

The comparison of the maximum extensional viscosity values displayed by the different lubricants tested reveals that *gels* (*i.e.*, Oralieve) present an extremely higher stretchiness (*η*_extensional_ = 2.0.10^3^ ± 1.3.10^3^ Pa s) as compared to that of the vegan lubricant (*η*_extensional_ = 72.0 ± 60.8 Pa s), BioXtra *viscous liquid* (*η*_extensional_ = 12.9 ± 4.8 Pa s), and the thinner Boots *liquid* (η_extensional_ = 5.2 ± 0.6 Pa s) (Fig. 5). Contrary to Boots *liquid*, which exhibits a high Trouton ratio (T_r_ = 289 ± 35) and thereby an elastic behaviour, slightly stronger than saliva, Oralieve *gel*, the vegan lubricant, and BioXtra *viscous liquid* display low *T*_*r*_ values (T_r_ = 8 ± 1, 12 ± 2, and 20 ± 9, respectively), which highlight the predominance of viscous forces over elasticity (Fig. 5).

Saliva is characterised by a high elasticity (*η*_*extensional*_ of up to ca. 0.6 Pa s) and a low shear viscosity, which results in a high Trouton ratio (*T*_*r*_ of up to ca. 120)^48^, therefore displaying strikingly higher viscoelastic properties than both the fabricated lubricants and the products tested, except Boots. Nonetheless, contrary to the other tested commercial samples, the fabricated, vegan lubricant shows a capillary break-up time very similar to that of human saliva, which has been reported to be of ca. t_b_ = 2 s^49^. Such a similar filament persistency is achieved by compensating the lower elasticity with a higher shear viscosity. Oralieve *gel*, which shows a very low elasticity and a much higher shear viscosity, brings this to the extreme and therefore display capillary break-up times 10 times higher than saliva.

## Discussion

Saliva substitutes currently available on the marketplace exhibit limited ability to alleviate patients’ symptoms^12,19^ offering short-lived relief from dryness sensation. To restore oral lubrication both efficiently and over a longer period, a commercial saliva substitute must provide three key functions that have been demonstrated to play a key role in salivary function: (1) high moisturisation, (2) strong binding, and (3) efficient lubricity. Many, if not most, commercial salivary substitutes focus on increasing viscosity through the use of hydrophilic thickening/gelling agents^17,18^—due to their lack of adsorption properties^19^—readily desorb from the hydrophobic oral mucosal surface and ultimately, lose their lubricating effect not long after being swallowed.

Herein, our aim was to assess and compare the dry mouth-hydrating capability of the novel, microgel-reinforced hydrogel-based aqueous lubricants we have recently fabricated in our lab^30^ showing better lubricity than human saliva, against a range of salivary replacers widely available on the marketplace and largely employed by xerostomia sufferers. For this purpose, their rheological (both viscous and extensional) and adsorption (on dry mouth-replicating surfaces) behaviours were investigated and linked to their ability to reduce friction under simulated oral dryness conditions. Two versions of the innovative aqueous lubricants, differing in their protein/polysaccharide composition (a dairy alternative made up of lactoferrin and *κ*-carrageenan, and a vegan one made up of potato protein and xanthan gum) (Fig. 1), were characterised, and eight saliva substitutes were selected based on their frequent use as described in a pilot (unpublished) focus group (11 dry mouth patients and 8 carers), type of lubricating agents they contain (carboxymethylcellulose, hydroxyethylcellulose, carbomer, xanthan gum, mucin), their format (spray, gel), and their wide presence on the UK, EU, and US markets (Table S1), for benchmarking purposes.

The super-lubricious human saliva^28,50,51^ exhibits particularly low shear viscosity, which clearly indicates that shear properties do not contribute to mouth moistening, even though certainly allowing the easy slide of food along the oral surfaces during food oral processing. While human saliva is poorly resistant to shear deformation, it displays a remarkable resistance to extensional flow, which has been demonstrated to assist the swallowing action^41–47^; nonetheless, its influence on chewing and speech articulation (if any) still needs to be elucidated. Although the role of the viscoelastic properties of human saliva in oral lubrication remains ambiguous, the resistance to shear (Fig. 2) and extensional (Fig. 5) deformation of each lubricant was benchmarked against existing commercial products in the marketplace. Both the fabricated aqueous lubricant and commercial products were found to be much more viscous than human saliva (*η*_shear_ > 15.10^–3^ Pa s for the samples studied against *η*_shear_ = 2.5.10^–3^ Pa s for human saliva^34^, at an orally relevant shear rate of 50 s^−1^) (Fig. 2). Compared to Oralieve gel, the vegan lubricant, and BioXtra *viscous liquid*, Boots *liquid* displayed a capillary thinning dynamics and filament shape dominated by elasticity (Fig. 5), but a lower filament persistency (*i.e.*, shorter breakage time) (Figure S6). Additionally, the vegan lubricant was found to be the only tested sample displaying a capillary break-up time comparable to that of human saliva (t_b_ = 1.63 ± 1.21 s *vs*. ca t_b_ = 2 s for human saliva^49^) (Figure S6), and this is achieved by a higher shear viscosity and lower elasticity compared to human saliva.

Friction, lubrication, and wear occur during motion of oral surfaces, such as the tongue and palate, and particularly arise during speech articulation and food oral processing—two activities rendered particularly difficult for dry mouth sufferers due to their lack of saliva. Soft tribology, which allows measuring the friction between two deformable surfaces in contact as a function of their motion, is an effective tool to assess the lubrication properties of any ingested products^38,52–54^. The primary property of a saliva-replacing product being to efficiently moisten oral mucosa and cavity, tribology measurements were carried out in the presence of dry mouth-mimicking surfaces (both PDMS and biomimetic tongue surfaces) differing in their surface topography and contact pressures in order to compare the lubrication performance of the fabricated aqueous lubricant against that of competing products (Fig. 3). This study demonstrates that the fabricated aqueous lubricant (both the dairy and vegan variants) displays extremely low friction coefficients in both the boundary and hydrodynamic regimes, thereby clearly revealing its high friction-reducing effect when in contact with dehydrated, smooth hydrophobic surfaces. In particular, the dairy protein-based lubricant formulation particularly offers better boundary lubricity compared to the range of commercial saliva replacers tested and the naturally lubricating human saliva in both PDMS and biomimetic tongue-like surface. In particular, tribology measurements with highly hydrophobic PDMS surfaces show that the fabricated aqueous lubricant outperforms both *liquid* and *viscous liquid* saliva substitutes in the boundary region (at 0.01 m s^−1^), by decreasing friction coefficients by 41–99%, and *gel* saliva substitutes in the hydrodynamic region (at 0.25 m s^−1^), by reducing friction coefficients by 83–89%. For biomimetic tongue-like textured surface, the key feature was the dairy lubricant offered lowest boundary friction as compared to all the tested products and showed an early onset of elastohydrodynamic regime at low speeds < 0.001 m s^−1^ similar to those of gels, despite having order of magnitude lower viscosity than the gels. Such dual benefits of the boundary and fluid film lubrication might be attributed to the patchiness of the microgel-reinforced hydrogels such that the uncovered proteinaceous microgels offered boundary lubrication and the hydrogel offered the hydrodynamic lift^30^. The discrepancy in behaviour of the two lubricant variants particularly in the biomimetic tongue-like surface might be attributed to the difference in adsorption behaviour of the protein types in surfaces with topographic features where the plant protein-based microgel variant demonstrated a tribological behaviour that resembled closely a diluted human saliva composition^30^, which did not show classic gel-like hydrodynamic behaviour.

Hydration studies were conducted to investigate the efficacy of each lubricant at adsorbing onto a dry tongue/palate proxy (PDMS) surface following ingestion, and at remaining attached following swallowing—these two properties being key to ensuring in vitro coating and consequently longevity (Fig. 4). Similarly, to human saliva, which was found to readily and lastingly adhere to an oral mucosa-simulating surface, a saliva-replacing product would need to exhibit a high binding capacity both before and after swallowing, to be effective at providing both lubricity-improving and long-lasting hydration. All salivary replacers, including the fabricated aqueous lubricant, showed strong mucoadhesive properties, except Glandosane (No Flavours) *liquid*, latter barely attached to the surface following injection; both *gels* (*i.e.*, Biotène and Oralieve) and the vegan version of the fabricated lubricant demonstrated a much stronger binding capacity than the other saliva substitutes tested. The particularly effective adsorption properties of the *gels* could be explained by their high viscosity leading to the formation of a thick, strongly cohesive hydrated layer. Additionally, both *liquids* (*i.e.*, Glandosane (No Flavours) and Boots) and *gels* (*i.e.*, Biotène and Oralieve) were shown to adsorb onto the surface at a much higher rate than the invented aqueous lubricants and BioXtra *viscous liquid*, which seemed to slowly diffuse towards the interface like real human saliva, most likely due to the protein content. This might be associated with these salivary substitute samples containing proteins (Table S1) that tend to adsorb at a slower rate.

Buffer rinsing (replicating the swallowing process) was found to have a relatively small impact on the adsorption of the fabricated aqueous lubricant (only 7%-layer removal for both the dairy and vegan alternatives) showing high in vitro coating indices, contrary to all commercial saliva substitutes that underwent 23–58% desorption. In other words, the fabricated technology remained attached to the surface 70–88% more than the tested products. All these results clearly demonstrate that the fabricated aqueous lubricant is the only one generating both strong adsorption and high retention, following a diffusion-controlled process, therefore replicating quite well human saliva mucoadhesive properties despite difference in rheological properties, and suggesting a better efficacy for providing long-lasting relief. The high rinsing-induced desorption observed with the commercial products tested could be attributed to the lack of hydrophobically binding molecules in their formulation; the lubricating agents they contain are hydrophilic polymers (carboxymethylcellulose, hydroxyethylcellulose, among others) that seem to not withstand buffer washing and to easily detach from the surface, thus correlating with the short-lasting relief period patients complain about.

## Limitations

Even though lubricity measurements were carried out under orally relevant conditions, a key limitation of this study is the lack of data regarding the long-term hydration efficiency of the fabricated lubricant *vs*. the competitive samples, which was not covered in this work. Although proteins such as lactoferrin have been previously shown to continuously adsorb due to intramolecular electrostatic interactions^29^, whether such an adsorption behaviour persists when lactoferrin is in a microgel form or embedded in a hydrogel structure is yet to be reported. Of more importance, such an assessment requires experimental work of several hours with and without subsequent exposure to pH and ions mimicking ingestion of food/beverages, which is beyond the scope of this study. Dynamic tribological measurements^55^ without any saliva addition could be combined with QCM-D experiments with repeated buffer rinsing to obtain a meaningful comparison of long-term hydration efficiency. Secondly, one might argue that the temperature of any ingested products might also negatively affect the lubrication properties of these designed aqueous lubricants. Since the microgels^56^ used in this work as the key boundary lubricants are irreversibly cross-linked via thermal treatment and ultimately non-temperature responsive, the impact of temperature on their lubrication performance following food/ beverage ingestion is likely to remain minimal. Nevertheless, a detailed study should be carried out in the future to confirm such a statement.

## Conclusions

Herein, we demonstrate that invented microgel-reinforced hydrogel formulation exhibits an outstanding and unprecedented capacity to drastically reduce friction between dehydrated oral surfaces under in vitro conditions, suggesting a potential to ultimately alleviate symptoms associated with dry mouth. The fabricated aqueous lubricant formulations, particularly the dairy protein type, were found to lubricate hydrophobic surfaces to a much higher and much longer extent than commercial saliva-replacing products particularly liquids and viscous liquids in boundary regime irrespective of the topography of the surface and outperform gels in a speed-dependent manner depending upon the surface used, offering up to 99% more effective lubrication and up to 88% stronger retention. Additionally, the fabricated formulation having similar capillary break time as that of saliva largely attributed to the viscous behaviour was shown to behave very similarly to real human saliva, exhibiting strong adhesion onto mucosa depleted-mimicking surfaces (*i.e.*, extensive adsorption and minimal desorption from dry oral-mimicking surfaces). The inefficient lubrication properties and short relief period displayed by currently marketed products are attributed to their inability to stick efficiently onto biological surfaces (human tongue and palate), in turn thought to be due to their lack of mucoadhesive molecules—whose importance in oral lubrication has been largely neglected until now. In contrast, the biocompatible aqueous lubricant provides both high moisturising capacity (long lasting hydration, thanks to the water-encapsulating biopolymeric hydrogel) and strong ability to stay on biological surfaces following ingestion (boundary lubrication, thanks to the efficiently adsorbing proteinaceous microgel). This robust proof-of-concept in vitro work is a first step towards shedding light on the high potential of microgel-based aqueous lubricants to work as a saliva substitute for dry mouth sufferers, and will certainly act as a springboard for future sensory evaluation and follow-up phase I clinical trials to confirm the subjective perception of moistness and real-world efficacy with dry mouth sufferers, respectively.

## Experimental section

### Materials

Lactoferrin (96.9% protein content) was purchased from Ingredia (Arras, France), potato protein isolate (91% protein content) from Sosa Ingredients (Barcelona, Spain), 4-(2-hydroxyethyl)-1-piperazineethanesulfonic acid (HEPES, *p* > 99.5%) from Illinois Tool Works Inc. (Panreac Quimica, Barcelona, Spain), citric acid monohydrate (*p* > 99.5%) from Alfa Aesar (Thermo Fisher Scientific, Lancashire, UK), Decon 90 from Decon Lab Ltd (Hove, UK), ammonia solution (25 wt%) and toluene from Fisher Scientific (Thermo Fisher Scientific Inc, Loughborough, UK), isopropanol (P99.8%) from MBFibreglass (Newtownabbey, UK), and *κ*-carrageenan, xanthan gum, trisodium citrate dihydrate, sodium hydroxide (NaOH, 1 M), hydrochloric acid (HCl, 1 M), sodium azide (NaN_3_, *p* > 99.5%), silicon oil, sulfuric acid (P95.0–98.0%), and hydrogen peroxide solution (30 wt%) from Sigma-Aldrich (Gillingham, UK). The Ecoflex™ 00–30 kit used to make the biomimetic tongue surface for the tribology measurements was bought from Smooth-on Inc. (Macungie, Pennsylvania, USA), and the two components were mixed at a 1:1 w/w ratio. The SYLGARD™ 184 silicone elastomer kit employed to coat the silicon sensors with polydimethylsiloxane (PDMS) for the quartz-crystal microbalance with dissipation monitoring (QCM-D) experiments was obtained from Dow Chemical Company Ltd (Cheadle, UK), and the silicon monomer and cross-linking agent were mixed at a 10:1 w/w ratio. Commercial saliva substitutes (*i.e.*, Glandosane (No flavours, Lemon flavour, and Peppermint flavour), sprays from Fresenius-Kabi; A.S Saliva Orthana, a spray from CCMed; Boots spray; Saliveze, a spray from Wyvern Medical; BioXtra, a viscous spray from RIS; Biotène, a gel from GSK; Aldiamed, a gel from Certmedica International; and Oralieve gel) were all purchased from common retailers (Table S1). Ultrapure water, or MilliQ-grade water (18.2 MΩ·cm, Merck Millipore, Bedford, MA, USA), was used in all experiments. HEPES buffer (pH 7.0) was prepared by dissolving 10 mM powdered HEPES in ultrapure water and adjusting the pH to salivary pH with 1 M NaOH. Citrate buffer (pH 5.0) was prepared by mixing 10 mM citric acid monohydrate and 10 mM trisodium citrate dihydrate in adequate proportions so as to reach the appropriate acidic pH. NaN_3_ (0.02 wt%) was added to all solutions as a preservative. All reagents were used as supplied without any further purification.

### Methods

#### Patented aqueous lubricant formulation preparation

Two aqueous lubricant formulations (for which patent was filed)^31^ were fabricated using two different protein types (lactoferrin and potato protein isolate) forming microgels of different sizes using the method described previously^30^; similar principles of proteinaceous microgelation and electrostatic coating of the microgels with oppositely charged polysaccharide hydrogels, were followed.

##### Dairy alternative

The dairy protein-based aqueous lubricant was prepared following a previously published protocol^30^. Briefly, lactoferrin solution (12 wt%) was prepared by adding powdered lactoferrin in 10 mM HEPES buffer at pH 7.0 and stirring for ca. 2 h to ensure complete solubilisation. Then, the solution was heated at 90 °C for 30 min to form a thermally cross-linked macroscopic gel via disulphide bonding, which was subsequently mixed with 10 mM HEPES buffer (3:1 w/w) at pH 7.0 and broken down into macrogel particles using a hand blender (HB724, Kenwood, Havant, UK), for 5 min. The resulting macrogel particles were degassed for 3 min with a conditioning mixer (ARE-250, THINKY Corporation, Tokyo, Japan), and finally passed twice through a bespoke Leeds Jet Homogeniser, at 300 ± 20 bars, to form lactoferrin microgel (LFM) particles. *κ*-carrageenan hydrogel (KCH, 1.5 wt%) was prepared via the dissolution of powdered *κ*-carrageenan in 10 mM HEPES buffer at pH 7.0 by heating at 95°C while being sheared for 30 min to ensure complete solubilisation. The aqueous solution was then cooled to around 37 °C, and LFM was added dropwise under gentle stirring, to form the aqueous lubricant formulation at a 0.6:1 w/w KCH/LFM ratio corresponding to a mixture of 1.2 wt% KCH and 2.0 wt% LFM, such ratio was particularly chosen to create a patchy architecture such that the lactoferrin microgel is not fully covered by the carrageenan hydrogel. This lactoferrin-containing aqueous lubricant is referred to as ‘dairy lubricant’.

##### Vegan alternative

Based upon the aforementioned technique, a vegan variant of the aqueous lubricant was fabricated using different pH, thermal treatment, and homogenisation conditions. Potato protein isolate solution (6.0 wt%) was prepared by adding powdered potato protein isolate in 10 mM citrate buffer at pH 5.0 and stirring for ca. 1.5 h to ensure complete solubilisation. Then, the pH of the solution was adjusted to 5.0 by adding 1 M HCl, and the solution was heated at 65 °C for 30 min to form potato protein microgel (PoPM). Xanthan gum hydrogel (XGH, 1.5 wt%) was prepared by dissolving powdered xanthan gum in 10 mM citrate buffer at pH 5.0 at 21 ± 2 °C and shearing the solution for 24 h under constant stirring for complete hydration. PoPM was added to XGH dropwise at 21 ± 2 °C, under gentle stirring, to form the aqueous lubricant formulation at a 0.5:1 w/w XGH/PoPM ratio corresponding to a mixture of 1.0 wt% XGH and 2.0 wt% PoPM to create the patchy architecture. This potato protein-containing aqueous lubricant is referred to as ‘vegan lubricant’.

#### Hydrodynamic diameter

Hydrodynamic diameters (*d*_H_) of the microgels *i.e.* LFM and PoPM were measured using dynamic light scattering with a Zetasizer Ultra (Malvern Instruments Ltd., Worcestershire, UK), at 25 °C. Size measurements were carried out following a 1:10 v/v dilution in the respective buffers (pH 7.0-HEPES buffer for the dairy lubricant and pH 5.0-citrate buffer for the vegan lubricant) using disposable cuvettes (ZEN0040), at an detection angle of 173.0°. The *d*_H_ results were reported as the mean value of at least nine readings made on triplicate samples.

#### Human saliva collection

Human saliva was collected from healthy subjects (n = 15) who were refrained from eating/drinking for at least 2 h before saliva collection and measurement. Subjects gave their written informed consent before taking part in the study with ethics approval from the University of Leeds (MEEC 16-046, ethics approved by the Faculty Ethics Committee, University of Leeds) in accordance with the relevant guidelines and regulations of the University of Leeds. Immediately after collection, saliva was centrifuged at 3000 g for 3 min and diluted 1:10 v/v with 10 mM HEPES buffer at pH 7.0. The supernatant was taken out and used for characterisation. Such centrifugation step is evidenced not to impair the lubrication performance of saliva^57^ but remove the interfering materials such as cells and debris. The rheological, lubrication and adsorption properties of human saliva were measured and used as controls for comparison purposes.

#### Rheological properties

##### Rotational rheometry

Resistance to shearing was assessed with a stress-controlled rheometer (Kinexus ultra + rotational rheometer, Malvern Instruments, Malvern, UK), fitted with a stainless-steel cone/plate geometry (2° angle cone/60 mm diameter (CP2/60) combined with a 65 mm diameter plate (PL65)) and equipped with a temperature-controlled Peltier system (with a ± 0.1 °C temperature stability at thermal equilibrium). Each sample (ca. 2 mL) was loaded onto the lower plate and overfill was correctly trimmed prior to adjusting the upper plate to a gap size of 0.70 mm. A thin layer of low viscosity silicone oil was deposited around the edges of the sample exposed to air, and a clamshell cover was placed over the system to prevent sample drying/evaporation and ensure temperature equilibration throughout the measurement. Apparent shear viscosity (${\eta }_{shear}$) was recorded over a shear rate ranging from 0.1 to 1000 s^−1^, at an orally relevant temperature of 37 °C. Each test was repeated at least three times on triplicate samples; the average measurement is shown.

##### Extensional rheometry

Resistance to stretching was measured using a HAAKE capillary breakup extensional rheometer (CaBER) 1 (Thermo Electron, Karlruhe, Germany). The thinning of the midpoint diameter of the capillary bridge generated by the rapid separation of two circular plates (diameter of D_o_ = 6 mm) that axially constrained the sample was recorded using a laser micrometre, with a beam thickness of 1 mm and a resolution of 20 μm. The initial separation (h_o_) between the two circular plates was set at 3 mm, leading to an initial aspect ratio (h_0_/D_0_) of 0.5. The final axial displacement (h_f_) was set at 10 mm in 50 ms to allow filament thinning. Each sample (ca. 0.1 mL) was injected between the plates using a 1 mL syringe. The experiment was triggered 60 s after loading the sample, to limit shear and temperature preconditioning effects. At least five repetitions were performed at 37 °C. High-speed videos of the experiments were also taken at 1,000 frames/s, using a PhantomV1612 high-speed camera (Vision Research, Wayne, NJ, USA), to record the shape evolution of the capillary thread. Due to the possible vertical displacement of the minimum filament diameter, the images acquired were processed using the ImageJ software to detect the filament interface, and compared to the data acquired with the laser micrometre.

Two different models were used to fit and interpret the experimentally observed filament thinning dynamics, depending on the nature of the salivary substitute under study^58^:

(i) For elastic fluids following an upper convected Maxwell model, with a characteristic time scale (λ_c_), the elastocapillary force balance predicts an exponential diameter decay in time^58^:

1 $${D}_{min}\left(t\right) \sim {e}^{-\frac{t}{3{\uplambda }_{c}}}$$

where *D*_min_ is the instantaneous minimum filament diameter.

(ii) For power law fluids:

2 $$D_{min} \left( t \right) = 2{ }\phi_{o} \frac{\sigma }{K} \left( {t_{b} - t} \right)^{n}$$

where $\phi_{o}$ is a prefactor equal to 0.142, *K* and *n* two power law model parameters, *t*_*b*_ the filament breakup time, and *σ* the surface tension of the sample. Surface tension measurements were performed in triplicates using the Wilhelmy plate method (Kruss ST10, KRÜSS GmbH, Hamburg, Germany), at 37 °C and minimum speed (0.5 mm min^−1^) to limit the influence of the shear generated between the sample and measuring plate.

The cylindrical elements of the samples at the axial mid-plane plate were subject to a strain (*ε*) expressed as:

3 $$\varepsilon =-2\mathrm{ln}\frac{{D}_{min}}{{\mathrm{D}}_{\mathrm{o}}}$$

The instantaneous strain rate ($\dot{\varepsilon }$) for a cylindrical element of fluid is given by:

4 $$\dot{\varepsilon }= \frac{-2}{{D}_{min}} \frac{{dD}_{min}}{dt}$$

The apparent extensional viscosity of the *liquid* (${\eta }_{extensional}$) can therefore be expressed as:

5 $${\eta }_{extensional}= \frac{2\sigma /{D}_{min}}{\dot{\varepsilon }}$$

The transient Trouton ratio (*T*_*r*_) was computed as the ratio between the apparent extensional and shear viscosity. In the literature, the Trouton ratio is usually computed by dividing the apparent extensional viscosity that depends on the elongation rate, by a constant, reference shear viscosity. In this study, however, no reference shear viscosity could clearly be identified and measured. Indeed, for most fluids, the range of shear rates investigated did not allow measuring a zero-shear viscosity. Furthermore, the wide range of rheological properties of the products studied resulted in a wide range of strain rates achieved during the extensional rheometry measurements. For these reasons, the following definition of the Trouton ratio was preferred:

6 $$\mathrm{Tr}(\dot{\varepsilon })= \frac{{\eta }_{extensional}(\dot{\varepsilon })}{{\eta }_{shear}(\dot{\varepsilon })}$$

where the dependence of shear rate on the strain rate has been considered at the denominator.

#### Tribological properties

##### Tribology using smooth PDMS surfaces

The oral friction-reducing effect was evaluated by tribology, using a conventional mini-traction machine (MTM2, PCS Instruments, London, UK) in combination with smooth hydrophobic elastomeric surfaces, *i.e.*, a PDMS ball (19 mm diameter) and disc (46 mm diameter) in a sliding/rolling motion, displaying a 50 nm surface roughness and 2.0 MPa Young’s modulus^59^. A constant normal force of 2.0 N, corresponding to a Hertzian contact pressure of ca. 200 kPa^53^, and a temperature of 37 °C were applied for tribological measurements.

The relative motion of the rolling and sliding surfaces is represented by the entrainment speed, which is the average of the ball and disc linear speeds at the contact point, the sliding/rolling ratio being fixed at 50% (*i.e.*, the contribution of both rolling and sliding to motion being defined as equal). The evolution of the friction coefficient (*μ*) was recorded over an entrainment speed (*U*) range of 0.0035–1.0 m s^−1^. Each experiment was repeated at least three times on triplicate samples; the average measurement is shown.

##### Tribology using 3D tongue-like biomimetic surfaces

The lubrication behaviour was assessed in the presence of more biologically relevant surfaces, using a tribological setup (Kinexus ultra + rotational rheometer, Malvern Instruments, Malvern, UK) equipped with a steel plate-on-plate geometry (50 nm diameters), whose lower plate included a 3D-textured, elastomeric surface emulating a real human dry tongue surface in terms of deformability (stiffness), topography (roughness), and wettability (hydrophobicity)^32^. This surface was created using Ecoflex™ 00–30, which exhibits a 130 kPa Young’s modulus (an order of magnitude lower than PDMS), and a 3D-printed mould that replicated the diameter and spatial distribution of fungiform and filiform papillae. The average values of contact pressure in the fungiform and filiform species in this biomimetic 3D tongue-like surface^32^ were 33.0 and 9.8 kPa, respectively, replicating the real tongue pressure that varies from 10 to 70 kPa^60,61^ in adults. The tribological contact was formed by the biomimetic tongue surface and the steel plate. Friction coefficients (*μ*) were calculated as follows:

7 $$\mu = \frac{M}{{{\text{R}} \cdot {\text{F}}_{{\text{N}}} }}$$

where *M* corresponds to the torque measured by the instrument, R the plate radius (R = 0.025 m), and F_N_ the normal force (F_N_ = 1.0 N). A sweep angular speed (*Ω*) range of 0.0050–1.2 s^−1^ was chosen to obtain entrainment speed (*U*) values ranging from 0.0001 to 0.03 mm s^−1^ (with *U* = *Ω*.R). Friction coefficients (*μ*) were monitored as a function of the entrainment speed (*U*). Each experiment was repeated at least three times; the average measurement is shown.

#### Adsorption properties

The capacity to adsorb onto a dry mouth-mimicking surface, replicating the ingestion process, and to remain attached following rinsing, replicating the swallowing process, was evaluated by using a QCM-D (Q-Sense E4 system, Biolin Scientific AB, Västra Frölunda, Sweden), equipped with PDMS-coated sensors.

##### PDMS coating of QCM-D silicon sensors

Silica-coated QCM-D sensors (QSX-303, Q-Sense, Biolin Scientific AB, Västra Frölunda, Sweden) were first treated by UV/Ozone for 15 min to generate hydrophilic surfaces, and then immersed into sulfuric acid for 1 h, before being sonicated twice in ultrapure water for 10 min and dried under nitrogen. The substrates were further cleaned by immersing them into an RCA silicon wafer cleaning solution (made up of 5:1:1 ultrapure water/ammonia/30% hydrogen peroxide) at 80 °C, for 15 min, to remove any remaining organic/insoluble impurities, and by subjecting them to three cycles of 10-min sonication in ultrapure water, before drying them again under nitrogen. Cleaned surfaces were spin-coated with PDMS (prepared in toluene at a concentration 0.5 wt%) at 5,000 RPM (with a 2,500 RPM/s acceleration), for 60 s, and finally placed under vacuum overnight, at 80 °C, to ensure efficient PDMS cross-linking.

##### QCM-D measurement

Prior to any measurement, PDMS-coated silicon substrates were thoroughly cleaned through sequential immersion in toluene (30 s), isopropanol (30 s), and ultrapure water (5 min), before rinsing them extensively with ultrapure water, and drying them under nitrogen.

Once assembled, the QCM-D flow cells were prefilled with either HEPES or citrate buffer until reaching a stable baseline. Each sample was diluted (*i.e.*, 20-fold dilution in HEPES buffer for commercial products, human saliva, and the dairy lubricant; 20-fold dilution in citrate buffer for the vegan lubricant) before being injected into the PDMS-coated silica sensor-containing cell. Once frequency and dissipation reached a plateau, characteristic of adsorption saturation, buffer was flushed into the cell, to study the desorption behaviour of the surface-adsorbed lubricant. Solutions were injected at a flow rate of 100 μL/min, and measurements conducted at a temperature of 25 ± 2 °C. Changes in resonance frequency (Δ*f*) were recorded simultaneously over time. Each experiment was reproduced at least three times; a representative curve is shown.

#### Statistical analyses

Statistical analysis was carried out with the GraphPad Prism software^62^, using the Kruskal–Wallis test, followed by the Dunn’s multiple comparisons test with a 95% confidence level, meaning that differences were considered as statistically significant when *p* < *0.05*.

### Supplementary Information

Supplementary Information.

## Data Availability

The data used to support the findings of this study are available from the corresponding author upon request.
