# Supplementary material for: Benchmarking of a microgel-reinforced hydrogel-based aqueous lubricant against commercial saliva substitutes
Source: Sci Rep. 2023 Nov 20;13:19833. doi: 10.1038/s41598-023-46108-w (PMC10662424; doi:10.1038/s41598-023-46108-w)
Supplement: Supplementary file 1 — Supplementary Information. [file 41598_2023_46108_MOESM1_ESM.docx]

**Benchmarking of a microgel-reinforced hydrogel-based aqueous lubricant against commercial saliva substitutes**

Olivia Pabois^a^, Alejandro Avila-Sierra^b^, Marco Ramaioli^b^, Mingduo Mu^a^, Yasmin Message^a^, Kwan-Mo You^a^, Evangelos Liamas^a, c^, Ben Kew^a^, Kalpana Durga^e^, Lisa Doherty^d^, Anwesha Sarkar^a*^

^a^ Food Colloids and Bioprocessing Group, School of Food Science and Nutrition, University of Leeds, Leeds LS2 9JT, United Kingdom

^b^ Université Paris-Saclay, INRAE, AgroParisTech, UMR SayFood, 91120 Palaiseau, France

^c^ Unilever Research & Development Port Sunlight Laboratory, Bebington CH63 3JW, United Kingdom

^d^ Vitrition UK Ltd, Liversedge WF15 6RA, United Kingdom

^e^ ADM Protexin Ltd, Lopen Head TA13 5JH, United Kingdom

E-mail addresses:

[o.pabois@leeds.ac.uk](mailto:o.pabois@leeds.ac.uk); [alejandro.avila-sierra@inrae.fr](mailto:alejandro.avila-sierra@inrae.fr); [marco.ramaioli@inrae.fr](mailto:marco.ramaioli@inrae.fr); [mingduo.mu@mail.mcgill.ca](mailto:mingduo.mu@mail.mcgill.ca); [ymessage@hotmail.co.uk](mailto:ymessage@hotmail.co.uk); [fskmy@leeds.ac.uk](mailto:fskmy@leeds.ac.uk); [evangelos.liamas@unilever.com](mailto:evangelos.liamas@unilever.com); [ll14bk@leeds.ac.uk](mailto:ll14bk@leeds.ac.uk); [kalpana.durga@adm.com](mailto:kalpana.durga@adm.com); [lisa.doherty@vituk.com](mailto:lisa.doherty@vituk.com); [a.sarkar@leeds.ac.uk](mailto:a.sarkar@leeds.ac.uk)

Corresponding author:

Prof. Anwesha Sarkar

Food Colloids and Bioprocessing Group

School of Food Science and Nutrition

University of Leeds

LS2 9JT Leeds, UK

**Supporting information**

**Table S1|** **Description of the commercial salivary replacers tested in this study.** Composition (with the key identified lubricating/thickening agent based on packaging information being underlined), pH, relief period (as stated on the packaging), and visual aspect of the commercial saliva substitutes selected for this benchmarking study.

|  | **Saliva substitute (manufacturer)** | **Composition**  **(lubricating/thickening agent)** | **pH** | **Relief period** |
| --- | --- | --- | --- | --- |
| 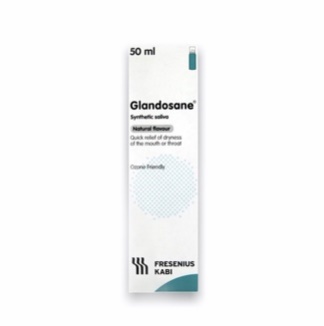 | **Glandosane**  **(Fresenius-Kabi)** | Sorbitol, Carboxymethylcellulose sodium, Potassium chloride, Sodium chloride, Sodium benzoate, Sorbic acid, Potassium monohydrogen phosphate, Calcium chloride, Magnesium chloride | 5.7 | N.S. |
| 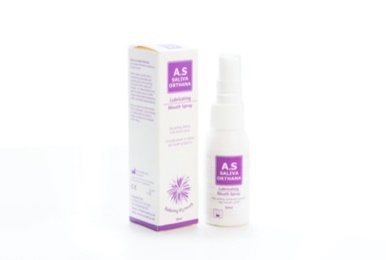 | **A.S Saliva Orthana**  **(CCMed)** | Mucin, Xylitol, Peppermint oil, Spearmint oil, Mineral salts, Methylparaben, Benzalkonium chloride, EDTA | 7.0 | N.S. |
| 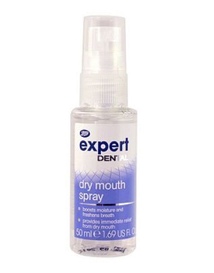 | **Boots** | Water, Glycerin, Xylitol, Sodium phosphate, Disodium phosphate, Polysorbate 20, Hydroxyethylcellulose, PEG-40 hydrogenated castor oil, Cetylpyridinium chloride, Sodium saccharin, Limonene | 6.5 | N.S. |
| 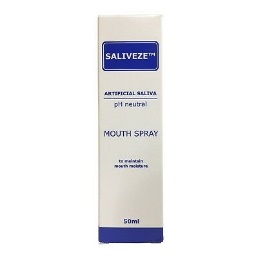 | **Saliveze**  **(Wyvern Medical)** | Calcium chloride, Magnesium chloride, Sodium chloride, Potassium chloride, Dibasic sodium phosphate, Carboxymethylcellulose, Sorbitol, Glycerol, Methylparaben, Propylparaben | 7.0 | N.S. |
| 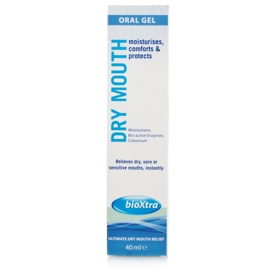 | **BioXtra**  **(RIS)** | Water, Xylitol, Hydrogenated starch hydrolysate, Sorbitol, Hydroxyethylcellulose, Sodium monofluorophosphate, Sodium saccharin, Potassium chloride, Sodium chloride, Magnesium chloride, Dipotassium phosphate, Calcium chloride, Colostrum whey, Lactoperoxidase, Citric acid, Sodium benzoate, Sodium methylparaben, Sodium propylparaben, Potassium sorbate | 7.0 | N.S. |
| 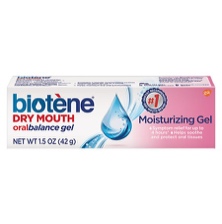 | **Biotène**  **(GSK)** | Glycerin, Water, Sorbitol, Xylitol, Carbomer, Hydroxyethylcellulose, Sodium hydroxide | 7.0 | up to 4h  (28-day  clinical study)^1^ |
| 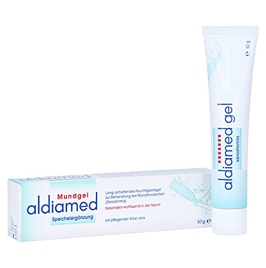 | **Aldiamed**  **(Certmedica International)** | Water, Glycerol, Sorbitol, Aloe Vera, Glyceryl polyacrylate, Lactoferrin, Lysozyme, Sodium hydroxide, Xanthan, Xylitol | 6.0 | hours |
| 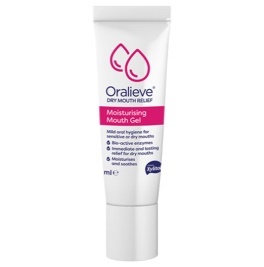 | **Oralieve** | Sorbitol, Glycerin, Water, Xylitol, Carbomer, Sorbic acid, Hydroxyethylcellulose, Whey protein, Sodium hydroxide, Benzoic acid, Glucose, Lactoferrin, Lactoperoxidase, Glucose oxidase, Potassium thiocyanate, Disodium phosphate, Aloe vera | 5.9 | 2-5 h (day),  up to 8 h (night) |

*Note: ‘N.S.’ stands for ‘Not Specified’.

**
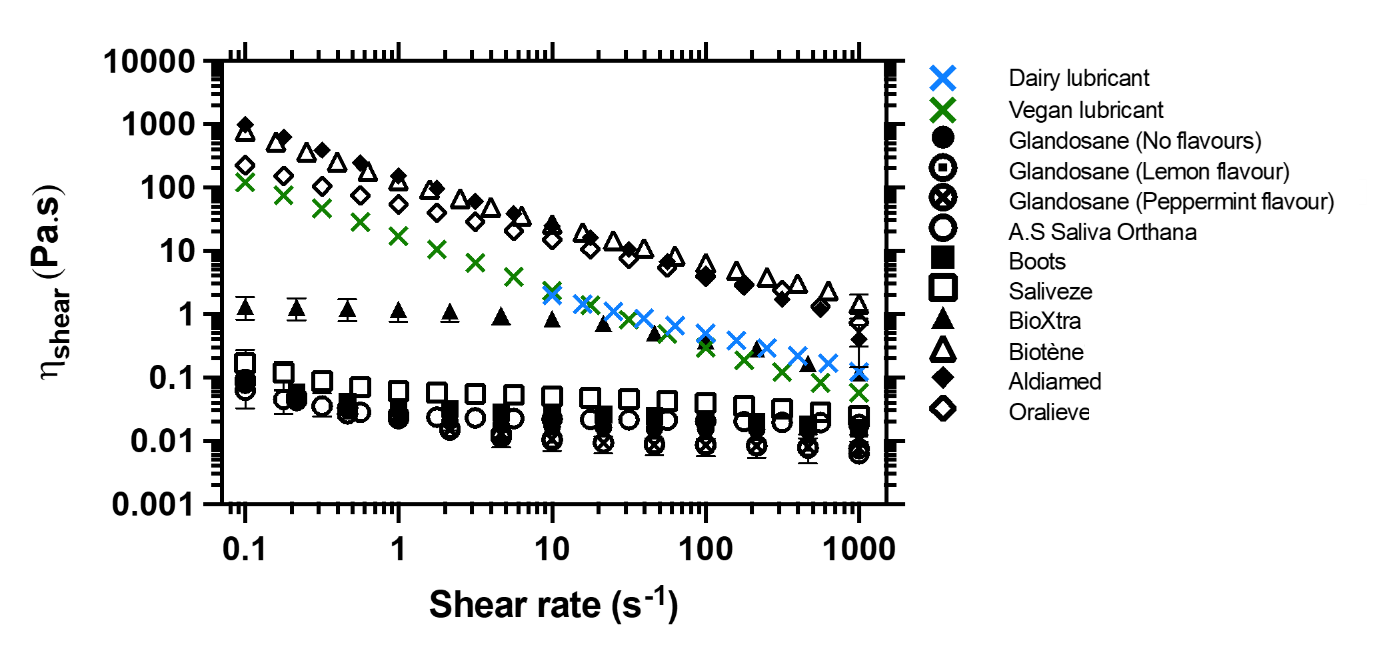
Figure S1|** **Flow curves of the fabricated aqueous lubricant benchmarked against commercial salivary replacers.** Evolution of the shear viscosity (*η_shear_*) as a function of the shear rate, obtained from stress-controlled rotational rheometry measurements performed on the fabricated aqueous lubricant (both dairy and vegan alternatives) and a range of commercially available saliva substitutes (*liquids*: Glandosane (No flavours, Lemon flavour, and Peppermint flavour) from Fresenius-Kabi, A.S Saliva Orthana from CCMed, Boots, and Saliveze from Wyvern Medical; *viscous liquids*: BioXtra from RIS; and *gels*: Biotène from GSK, Aldiamed from Certmedica International, and Oralieve), at an orally relevant temperature (37 °C). Each experiment was reproduced at least three times; the average measurement is shown with error bars representing standard deviations. The fabricated aqueous lubricants show a shear-thinning behaviour, with a trend similar to that of the commercial *gels*, but a viscosity value at an orally relevant shear rate (50 s^-1^) comparable to that of BioXtra *viscous liquid*.

**
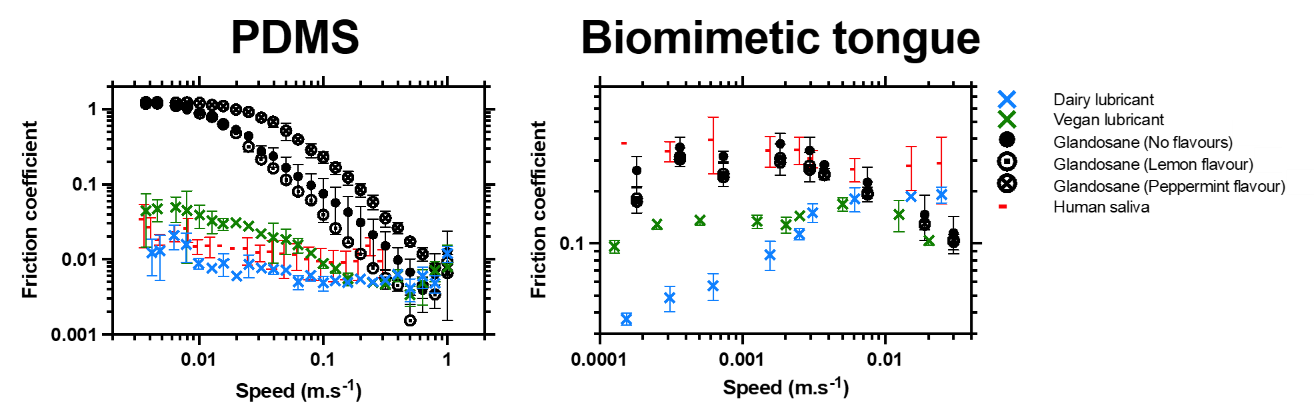
Figure S2| Lubrication performance of the fabricated aqueous lubricants benchmarked against a range of commercial *liquid* salivary replacers (sprays) under orally relevant conditions.** Speed-dependent evolution of the friction coefficient, obtained from tribology measurements performed with **smooth PDMS**, and **3D-textured, biomimetic tongue-like surfaces** replicating a dry mouth, on the fabricated aqueous lubricant (both vegan and dairy alternatives) and a range of commercially available *liquid* saliva substitutes (Glandosane (No flavours, Lemon flavour, and Peppermint flavour) from Fresenius-Kabi), at an orally relevant temperature (37 °C). The lubrication properties of real human saliva after centrifugation and dilution with 10 mM HEPES buffer (1: 10 v/v) at pH 7.0 are also shown and used as controls for comparison purposes (MEEC 16-046 ethics approved by the Faculty Ethics Committee, University of Leeds, UK). Each measurement was reproduced at least two times; the average measurement is shown with error bars representing standard deviations. Both the fabricated aqueous lubricants show an outstanding lubrication performance with orders of magnitude lower coefficients of friction than the commercial *liquid* samples in the boundary regimes in presence of highly hydrophobic PDMS surfaces. The dairy lubricant also exhibited much lower friction coefficients than the commercial *liquid* samples, particularly in the boundary regime.


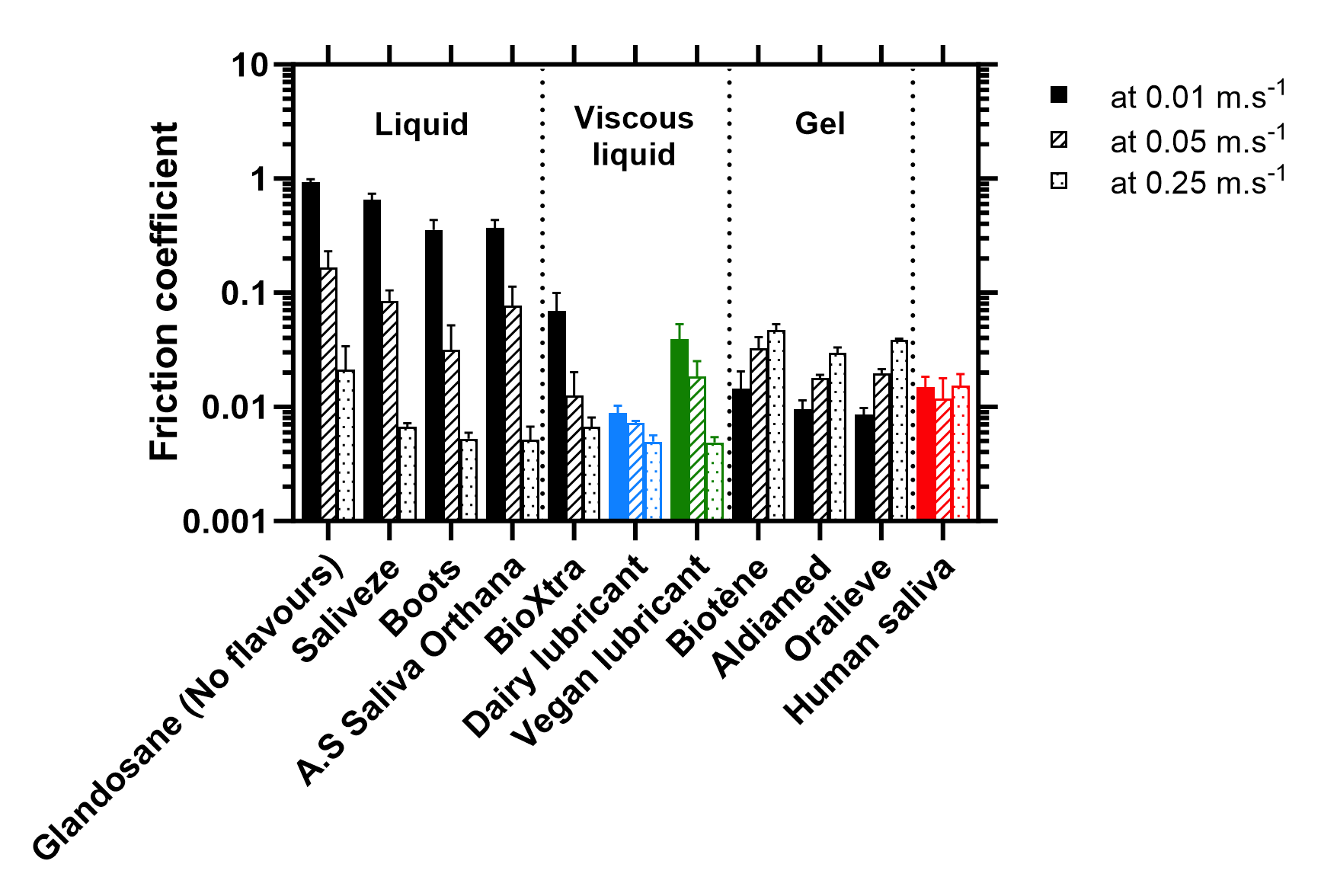


**Figure S3| Speed-dependent lubrication properties of the fabricated aqueous lubricant benchmarked against commercial salivary replacers using PDMS surfaces as tribopairs.** Comparison of the friction coefficients obtained at different entrainment speeds characteristic of the boundary (0.0007 and 0.01 m.s^-1^), mixed (0.02 and 0.05 m.s^-1^) and hydrodynamic (0.25 m.s^-1^) regimes, obtained from tribology measurements performed with smooth PDMS for the fabricated aqueous lubricant (both vegan and dairy alternatives) and a range of commercially available saliva substitutes (*liquids*: Glandosane (No flavours) from Fresenius-Kabi, Saliveze from Wyvern Medical, Boots, and A.S Saliva Orthana from CCMed; *viscous liquids*: BioXtra from RIS; and *gels*: Biotène from GSK, Aldiamed from Certmedica International, and Oralieve), at an orally relevant temperature (37 °C). Data corresponding to real human saliva are also shown and used as controls for comparison purposes (MEEC 16-046 ethics approved by the Faculty Ethics Committee, University of Leeds). These data were extracted from the tribology measurements (Figure 3). Each measurement was reproduced at least three times; the average measurement is shown with error bars representing standard deviations. The fabricated aqueous lubricants show an outstanding lubrication performance in the boundary regimes, exhibiting much lower friction coefficients than the commercial *liquid* and *viscous liquid* samples. The fabricated lubricants also show lower friction coefficients than commercial *gel* samples in the hydrodynamic regime. The lubricity of dairy lubricant is higher than that of real human saliva irrespective of speed (*p < 0.05*). The vegan lubricant shows friction equivalent to that of saliva (*p > 0.05*) in low-to-medium speeds (0.01-0.05 m.s^-1^), but lower friction than saliva in the higher speeds (0.25 m.s^-1^) (*p < 0.05*). In addition, the vegan lubricant shows sporadic behaviour with higher friction than gels in lowest speeds (0.001 m.s^-1^) (*p < 0.05*), equivalent friction to gels in medium speeds (0.05 m.s^-1^) (*p < 0.05*), but lower friction than gels in the higher speeds (0.25 m.s^-1^) (*p > 0.05*).


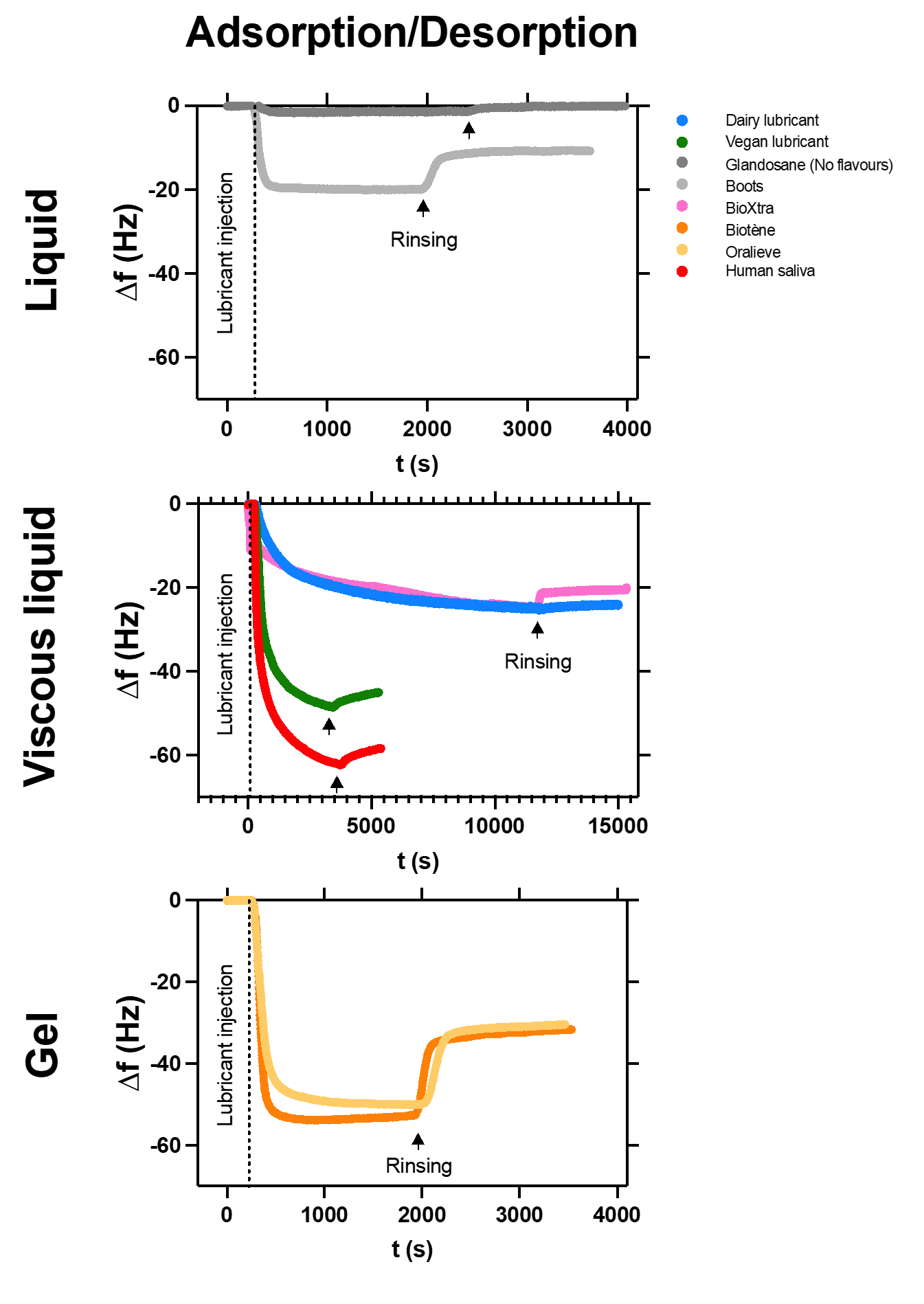


**Figure S4| Adsorption/desorption behaviour of the fabricated aqueous lubricant benchmarked against commercial salivary replacers, in the presence of a dry mouth-mimicking surface.** Time-dependent evolution of the resonance frequency (Δ*f*) measured using QCM-D, upon adsorption of the fabricated aqueous lubricant (both vegan and dairy alternatives) and a reduced range of commercially available saliva substitutes: ***liquids*:** Glandosane (No flavours) from Fresenius-Kabi, and Boots; ***viscous liquids*:** BioXtra from RIS; and ***gels*:** Biotène from GSK, and Oralieve), onto a dry mouth-replicating, PDMS-coated surface. Each lubricant was injected into the chamber, which was then rinsed with buffer to assess the sample ability to remain adsorbed to the surface following buffer rinsing. The adsorption properties of real human saliva are also shown and used as controls for comparison purposes (MEEC 16-046 ethics approved by the Faculty Ethics Committee, University of Leeds). For readability purposes, resonance frequencies are only shown for the 5^th^ overtone. Each measurement was reproduced at least three times; a representative curve is shown. The fabricated aqueous lubricants are able to both readily adsorb but at a slower rate as compared to the gels at the interface and remain strongly attached following rinsing, contrary to the studied commercially available saliva substitutes, which significantly desorb from the interface .

**
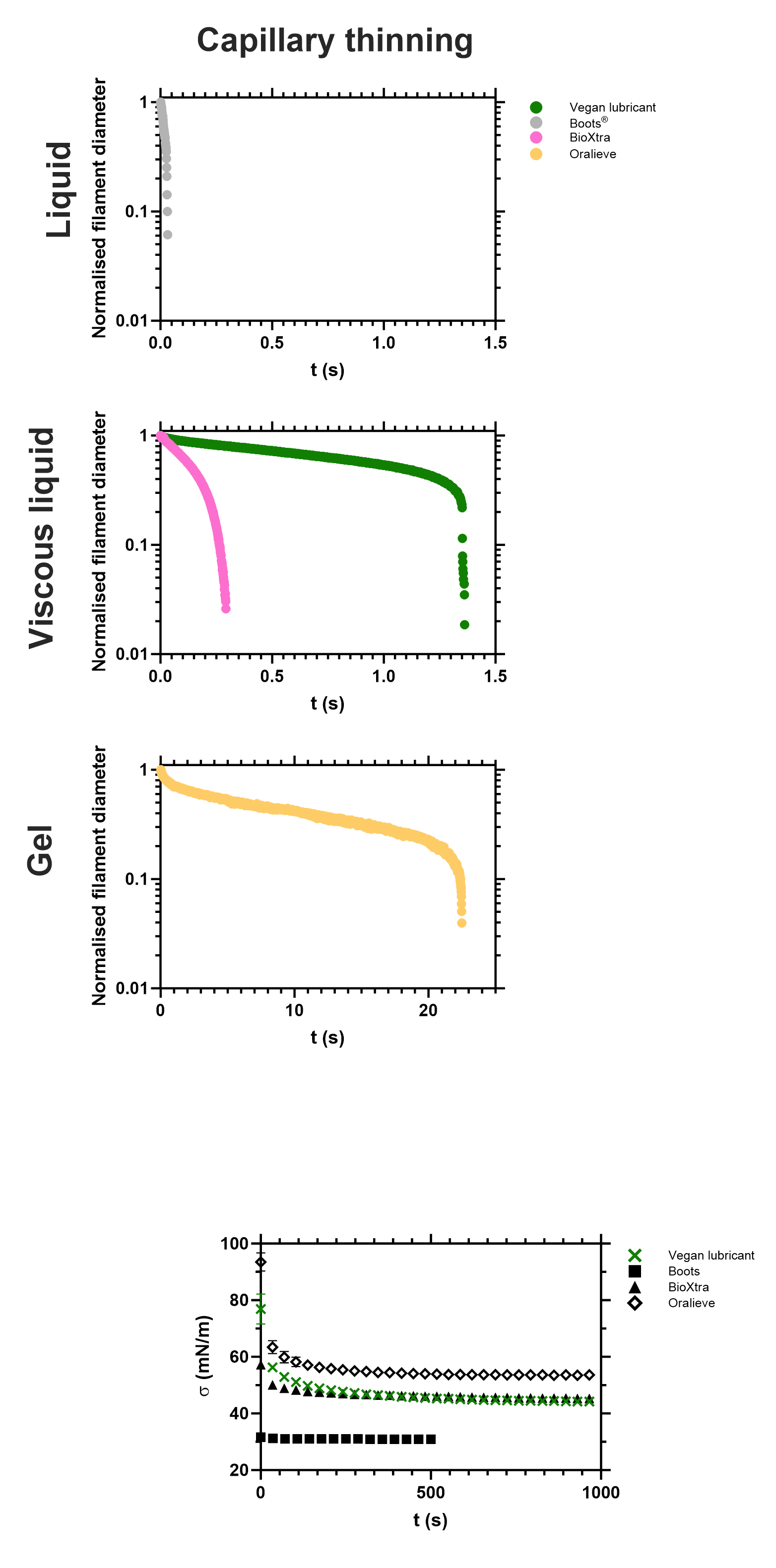
**

**Figure S5| Surface tension of the fabricated vegan aqueous lubricant benchmarked against commercial salivary replacers.** Time-dependent evolution of the surface tension (*σ*) of the fabricated aqueous lubricant (vegan alternative) and a reduced range of commercially available saliva substitutes (*liquid*: Boots; *viscous liquid*: BioXtra from RIS; and *gel*: Oralieve), measured with a Wilhelmy plate tensiometer, at an orally relevant temperature (37°C). Each measurement was reproduced at least three times; the average measurement is shown with error bars representing standard deviations. BioXtra *viscous liquid* and the fabricated vegan lubricant exhibit similar surface tension values (*i.e.*, σ = 45.2 ± 0.2 mN.m^-1^ and σ = 44.2 ± 0.1 mN.m^-1^, respectively), whereas Boots *liquid* and Oralieve *gel* display the lowest (σ = 30.9 ± 0.1 mN.m^-1^) and highest (σ = 53.6 ± 0.6 mN.m^-1^) surface activities, respectively. In comparison, human saliva shows a lower surface tension (ca. σ = 24.8 mN.m^-1^)^2^.

**
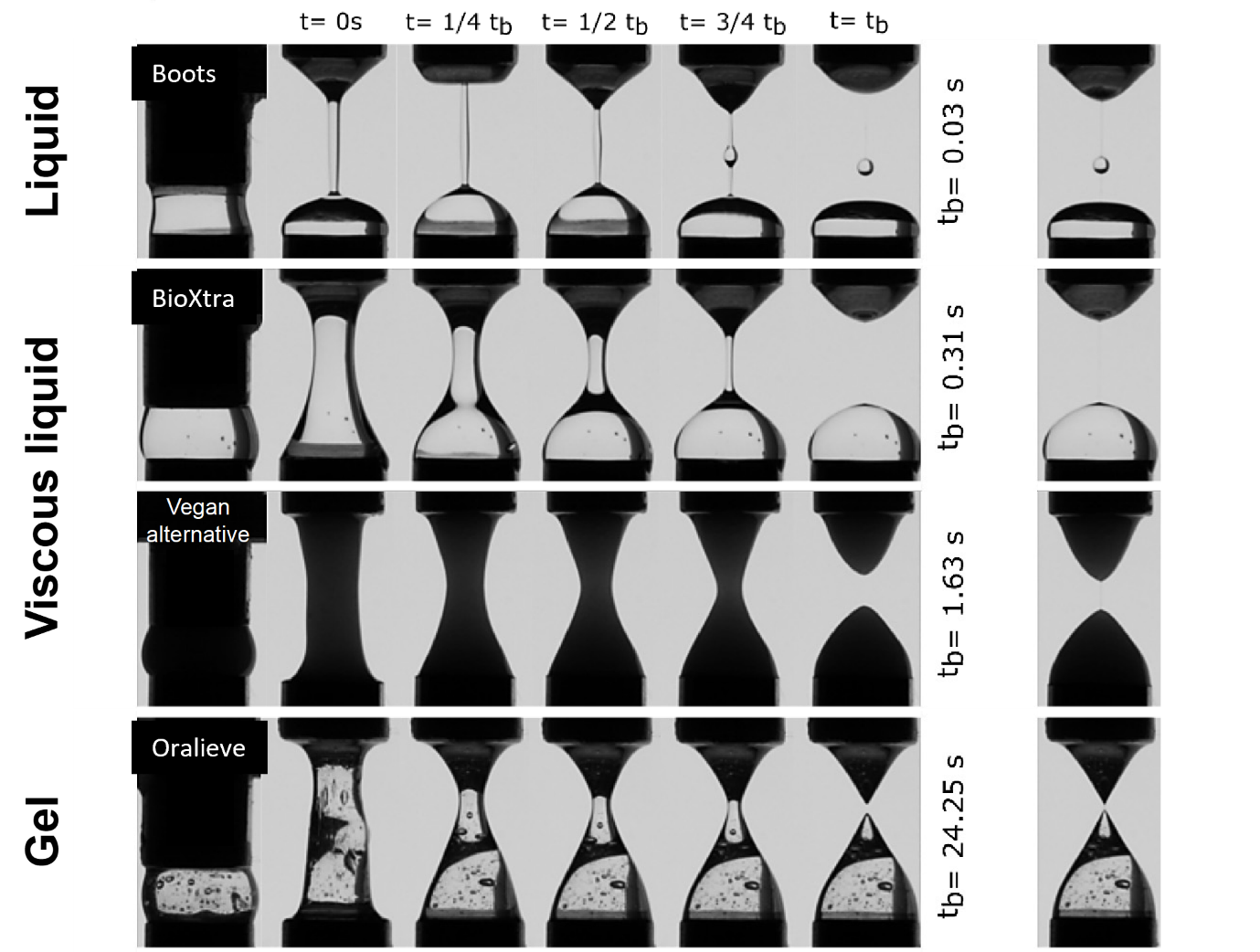
 Figure S6| Capillary thinning properties of the fabricated vegan aqueous lubricant benchmarked against commercial salivary replacers.** Stretching-induced capillary thinning behaviour observed over time with an extensional rheometer, for the fabricated aqueous lubricant (vegan alternative) and a reduced range of commercially available saliva substitutes: ***liquid*:** Boots; ***viscous liquids*:** BioXtra from RIS; and ***gel***: Oralieve), at an orally relevant temperature (37 °C). Times normalised with respect to the capillary break-up time (*t_b_*) are indicated, and an extra column on the right shows the shape of the filament just before break-up. Each experiment was reproduced at least three times; a representative measurement is shown. Similarly to BioXtra *viscous liquid*, for which t_b_ = 0.31 ± 0.01 s, the fabricated vegan lubricant filament does not break up before t_b_ = 1.63 ± 1.21 s, thus being more resistant to thread thinning than Boots *liquid* (t_b_ = 0.03 ± 0.00 s), and less than Oralieve *gel* (t_b_ = 24.25 ± 13.72 s). In comparison, human saliva shows a capillary break-up time of ca. t_b_ = 2 s^3^.

**
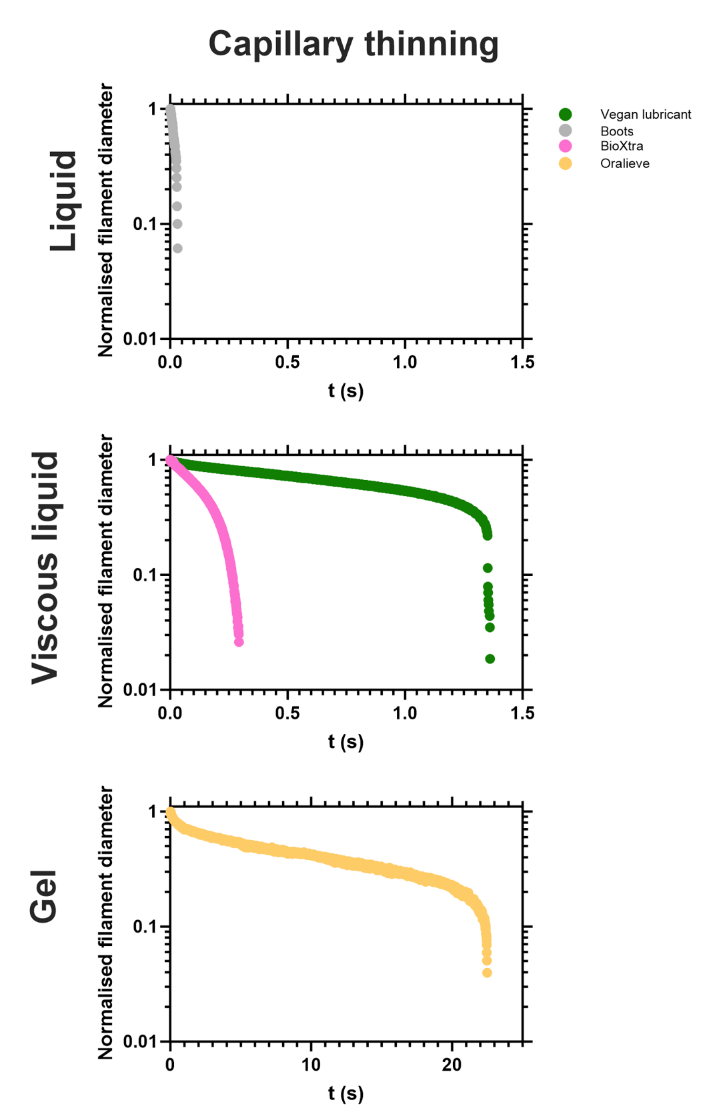
**

**Figure S7| Typical filament thinning dynamics observed for the fabricated vegan aqueous lubricant benchmarked against commercial salivary replacers with capillary breakage extensional rheometry.** Time-dependent evolution of the normalised filament diameter upon stretching, measured by extensional rheometry, for the fabricated aqueous lubricant (vegan alternative) and a reduced range of commercially available saliva substitutes: ***liquid*:** Boots; ***viscous liquids*:** BioXtra from RIS; ***gel*:** Oralieve, at an orally relevant temperature (37 °C). Each experiment was reproduced at least three times; a representative measurement is shown. All the samples exhibit a two-stage filament thinning mechanism, with (i) an initial, exponential regime, where a long thread forms, (ii) followed by a fast and marked exponential decay rapidly evolving into an axially uniform thin filament, eventually breaking up. *Viscous liquids*, including the fabricated vegan lubricant, show a higher resistance to capillary thinning than *liquids*, but a lower one compared to *gels*.

**References**

1 GlaxoSmithKline. *A clinical study to evaluate the efficacy of three dry mouth relief products versus water*, <<https://clinicaltrials.gov/ct2/show/NCT03494985>> (2018).

2 Preetha, A. & Banerjee, R. Comparison of artificial saliva substitutes. *Trends in biomaterials & artificial organs* **18** (2005).

3 Gardner, A., So, P.-W. & Carpenter, G. Endogenous salivary citrate is associated with enhanced rheological properties following oral capsaicin stimulation. *Experimental Physiology* **105**, 96-107, doi:10.1113/EP088166 (2020).
